# Polymorphisms in Stromal Genes and Susceptibility to Serous Epithelial Ovarian Cancer: A Report from the Ovarian Cancer Association Consortium

**DOI:** 10.1371/journal.pone.0019642

**Published:** 2011-05-27

**Authors:** Ernest K. Amankwah, Qinggang Wang, Joellen M. Schildkraut, Ya-Yu Tsai, Susan J. Ramus, Brooke L. Fridley, Jonathan Beesley, Sharon E. Johnatty, Penelope M. Webb, Georgia Chenevix-Trench, Australian Ovarian Cancer Study Group, Laura C. Dale, Diether Lambrechts, Frederic Amant, Evelyn Despierre, Ignace Vergote, Simon A. Gayther, Aleksandra Gentry-Maharaj, Usha Menon, Jenny Chang-Claude, Shan Wang-Gohrke, Hoda Anton-Culver, Argyrios Ziogas, Thilo Dörk, Matthias Dürst, Natalia Antonenkova, Natalia Bogdanova, Robert Brown, James M. Flanagan, Stanley B. Kaye, James Paul, Ralf Bützow, Heli Nevanlinna, Ian Campbell, Diana M. Eccles, Beth Y. Karlan, Jenny Gross, Christine Walsh, Paul D. P. Pharoah, Honglin Song, Susanne Krüger Kjær, Estrid Høgdall, Claus Høgdall, Lene Lundvall, Lotte Nedergaard, Lambertus A. L. M. Kiemeney, Leon F. A. G. Massuger, Anne M. van Altena, Sita H. H. M. Vermeulen, Nhu D. Le, Angela Brooks-Wilson, Linda S. Cook, Catherine M. Phelan, Julie M. Cunningham, Celine M. Vachon, Robert A. Vierkant, Edwin S. Iversen, Andrew Berchuck, Ellen L. Goode, Thomas A. Sellers, Linda E. Kelemen

**Affiliations:** 1 Department of Population Health Research, Alberta Health Services-Cancer Care, Calgary, Alberta, Canada; 2 Department of Community and Family Medicine, Duke University Medical Center, Durham, North Carolina, United States of America; 3 Division of Cancer Prevention and Control, H. Lee Moffitt Cancer Center and Research Institute, Tampa, Florida, United States of America; 4 Department of Gynaecological Oncology, UCL EGA Institute for Women's Health, University College London, London, United Kingdom; 5 Department of Health Sciences Research, Mayo Clinic, Rochester, Minnesota, United States of America; 6 The Queensland Institute of Medical Research, Post Office Royal Brisbane Hospital, Australia; 7 Peter MacCallum Cancer Centre, Melbourne, Australia; 8 Vesalius Research Center, VIB and KU Leuven, Leuven, Belgium; 9 Department of Obstetrics and Gynecology, University Hospitals Leuven, Leuven, Belgium; 10 Division of Cancer Epidemiology, German Cancer Research Center, Heidelberg, Germany; 11 Department of Obstetrics and Gynecology, University of Ulm, Ulm, Germany; 12 Department of Epidemiology, School of Medicine, University of California Irvine, Irvine, California, United States of America; 13 Clinics of Obstetrics and Gynaecology, Hannover Medical School, Hannover, Germany; 14 Department of Gynaecology, Jena University Hospital, Jena, Germany; 15 Byelorussian Institute for Oncology and Medical Radiology Aleksandrov N.N., Minsk, Belarus; 16 Epigenetics Unit, Department of Surgery and Cancer, Imperial College London, London, United Kingdom; 17 Section of Medicine, Institute Cancer Research, Sutton, United Kingdom; 18 The Beatson West of Scotland Cancer Centre, Glasgow University, Glasgow, United Kingdom; 19 Department of Pathology, University of Helsinki, Haartman Insitute, Helsinki, Finland; 20 Department of Obstetrics and Gynecology, Helsinki University, Central Hospital, Helsinki, Finland; 21 Department of Pathology, University of Melbourne, Parkville, Australia; 22 Wessex Clinical Genetics Service, Princess Anne Hospital, Southampton, United Kingdom; 23 Women's Cancer Research Institute at the Samuel Oschin Comprehensive Cancer Institute, Cedars-Sinai Medical Center, Los Angeles, California, United States of America; 24 Strangeways Research Laboratory, Cancer Research United Kingdom, Department of Oncology, University of Cambridge, Cambridge, United Kingdom; 25 Danish Cancer Society, Copenhagen, Denmark; 26 Gynecologic Clinic, Rigshospitalet, University of Copenhagen, Copenhagen, Denmark; 27 Danish Cancer Biobank, Copenhagen and Department of Pathology, Herlev Hospital, University of Copenhagen, Copenhagen, Denmark; 28 Department of Pathology, Rigshospitalet, University of Copenhagen, Copenhagen, Denmark; 29 Department of Epidemiology, Biostatistics, and Health Technology Assessment, Radboud University Nijmegen Medical Centre, Nijmegen, The Netherlands; 30 Department of Obstetrics and Gynaecology, Radboud University Nijmegen Medical Centre, Nijmegen, The Netherlands; 31 Cancer Control Research, British Columbia Cancer Agency, Vancouver, British Columbia, Canada; 32 Genome Sciences Centre, British Columbia Cancer Agency, Vancouver, British Columbia, Canada; 33 Department of Biomedical Physiology and Kinesiology, Simon Fraser University, Burnaby, British Columbia, Canada; 34 Division of Epidemiology and Biostatistics, University of New Mexico, Albuquerque, New Mexico, United States of America; 35 Department of Pathology and Laboratory Medicine, Mayo Clinic, Rochester, Minnesota, United States of America; 36 Departments of Oncology and Medical Genetics, University of Calgary, Calgary, Alberta, Canada; Univesity of Texas Southwestern Medical Center at Dallas, United States of America

## Abstract

Alterations in stromal tissue components can inhibit or promote epithelial tumorigenesis. Decorin (*DCN*) and lumican (*LUM*) show reduced stromal expression in serous epithelial ovarian cancer (sEOC). We hypothesized that common variants in these genes associate with risk. Associations with sEOC among Caucasians were estimated with odds ratios (OR) among 397 cases and 920 controls in two U.S.-based studies (discovery set), 436 cases and 1,098 controls in Australia (replication set 1) and a consortium of 15 studies comprising 1,668 cases and 4,249 controls (replication set 2). The discovery set and replication set 1 (833 cases and 2,013 controls) showed statistically homogeneous (P_heterogeneity_≥0.48) decreased risks of sEOC at four variants: *DCN* rs3138165, rs13312816 and rs516115, and *LUM* rs17018765 (OR = 0.6 to 0.9; P_trend_ = 0.001 to 0.03). Results from replication set 2 were statistically homogeneous (P_heterogeneity_≥0.13) and associated with increased risks at *DCN* rs3138165 and rs13312816, and *LUM* rs17018765: all ORs = 1.2; P_trend_≤0.02. The ORs at the four variants were statistically heterogeneous across all 18 studies (P_heterogeneity_≤0.03), which precluded combining. In post-hoc analyses, interactions were observed between each variant and recruitment period (P_interaction_≤0.003), age at diagnosis (P_interaction_ = 0.04), and year of diagnosis (P_interaction_ = 0.05) in the five studies with available information (1,044 cases, 2,469 controls). We conclude that variants in *DCN* and *LUM* are not directly associated with sEOC, and that confirmation of possible effect modification of the variants by non-genetic factors is required.

## Introduction

Cancers at the ovary are the most lethal gynecologic cancer, with 21,650 new cases and 15,520 deaths in the U.S. in 2008 [1]. Most (>95%) ovarian cancers are epithelial in origin, affecting cells on the surface of the ovary [2], which are separated from the underlying ovarian stromal tissue by a basal lamina. The stroma is the supportive framework of biologic tissue consisting of an extracellular matrix (ECM) and soluble growth factors that mediate epithelial-stromal interactions and regulate intercellular communication [3]. Activation of oncogenes and inhibition of tumor suppressor genes in the epithelium were previously considered to be the only alterations required for the development of epithelial cancers [4]; however, alterations in stromal components that disrupt normal cell functions can lead to morphologic changes that manifest as tumors through perturbation of the epithelium [3]. For example, radiation-induced changes in the stromal microenvironment have been shown to contribute to neoplastic progression of initiated mammary epithelial cells *in vivo* [5], and may include processes that activate transforming growth factor-beta (TGF-β) and initiate ECM remodelling [6], [7].

The ECM is composed of different proteins: decorin and lumican are members of the small leucine-rich proteoglycan family that bind to collagen in the stroma and are involved in matrix assembly and structure, and in the control of cell proliferation [8]. The expression of both decorin and lumican is altered in various cancers [9], [10], including serous epithelial ovarian cancer [11]–[14]. Conceivably, factors that alter epithelial-stromal interactions or the cross-talk among growth factors like TGF-β may also influence expression and/or activity of decorin or lumican, or vice versa. Such factors may include inherited genetic susceptibility. This could be particularly germane to decorin, which binds to TGF-β and serves as a regulatory control for TGF-β release and activation [15].

In view of the important role of the stroma in epithelial cancers and the role of decorin and lumican in tumorigenesis, we tested the hypothesis that inherited variation in *DCN* and *LUM* may influence the risk of serous epithelial ovarian cancer in 18 independent study populations: a discovery set that included studies from Mayo Clinic (MAY) and the North Carolina Ovarian Cancer (NCO) study, replication set 1 from Australia (AUS), and replication set 2 comprised of 12 matched studies from the Ovarian Cancer Association Consortium (OCAC).

## Results

The distributions of selected covariates between cases and controls in the discovery set and replication set 1 are listed in Table 1. Covariates were distributed similarly between the discovery set and replication set 1, including the proportion of serous carcinomas across tumor stage. The MAFs for the 10 tagSNPs in the discovery set ranged from 0.08 to 0.29 among controls and were similar in replication set 1 for those SNPs in common (Table S1).

**Table 1:** Distribution^A^ of selected characteristics between cases and controls.

	Discovery Set (MAY-NCO)					Replication Set 1 (AUS)			
Characteristic	Cases		Controls		Characteristic	Cases		Controls	
N	397		920		N	436		1098	
Age, yr [mean (SD)]	59.9	(11.2)	57.2	(12.7)	Age, yr [mean (SD)]	59.9	(9.95)	57.2	(11.8)
Age at menarche, yr					Age at menarche, yr				
<12	83	(23.6)	152	(16.8)	<12	77	(17.9)	192	(17.9)
12	84	(23.9)	239	(26.4)	12	102	(23.7)	234	(21.8)
13	97	(27.6)	266	(29.3)	13	109	(25.4)	289	(23.9)
≥14	88	(25.0)	250	(27.6)	≥14	142	(33.0)	358	(33.4)
Oral contraceptive use, mo					Oral contraceptive use				
Never	168	(43.6)	314	(34.7)	No	143	(32.8)	219	(20.0)
1–48	108	(28.1)	223	(24.6)	Yes	292	(67.0)	879	(80.1)
≥48	109	(28.3)	368	(40.7)					
Parity, n/age at first birth, yr					Full-term births, n				
Nulliparous	64	(16.1)	128	(14.0)	0	61	(14.0)	117	(10.7)
1–2/≤20	42	(10.6)	81	(8.8)	1	45	(10.3)	91	(8.3)
1–2/≥20	127	(32.0)	341	(37.2)	2	124	(28.5)	359	(32.7)
≥3/≤20	76	(19.1)	124	(13.5)	3	109	(25.1)	311	(28.3)
≥3/≥20	88	(22.2)	242	(26.4)	>3	96	(27.1)	220	(20.0)
Tumor stage					Tumor stage				
I	26	(6.7)			I	27	(6.5)		
II	21	(5.4)			II	26	(6.2)		
III	282	(72.5)			III	320	(76.7)		
IV	60	(15.4)			IV	44	(10.6)		

A Data are counts (%) except for age. Sample is 1,317 Caucasian subjects in the discovery set and 1,534 Caucasian subjects in replication set 1.

In the discovery set, decreased risks were associated with serous epithelial ovarian cancer under both co-dominant and ordinal models at *DCN* rs3138165, *DCN* rs13312816, *DCN* rs516115 and *LUM* rs17018765 (all four SNPs: P_trend_ = 0.06) (Table 2). Associations at all SNPs interrogated in the discovery set are in Table S2. No statistically significant associations were found in haplotype analyses (Table S3).

**Table 2:** Odds ratios (OR) and 95% confidence intervals (CI)^A^ between variants in *DCN* and *LUM* genes and serous epithelial ovarian cancer risk.

		Discovery Set			Replication Set 1			Combined Set		
		MAY-NCO			AUS			MAY-NCO and AUS		
Gene/SNP rsID	Genotype	Case/control	OR (95%CI)	P	Case/control	OR (95%CI)	P	Case/control	OR (95%CI)	P
*DCN*										
rs3138165^B^										
	GG	353/778	1.0 (Ref)		395/939	1.0 (Ref)		748/1717	1.0 (Ref)	
	GA	43/138	0.7 (0.5–1.0)		40/152	0.6 (0.4–0.9)		83/290	0.7 (0.5–0.9)	
	AA	1/4	0.7 (0.1–6.2)	0.17^D^	1/7	0.4 (0.0–3.3)	0.03^D^	2/11	0.5 (0.1–2.1)	0.006^D^
	Per allele		0.7 (0.5–1.0)	0.06^E^		0.6 (0.4–0.9)	0.009^E^		0.7 (0.5–0.9)	0.002^E^
rs13312816^C^										
	AA	353/778	1.0 (Ref)		389/928	1.0 (Ref)		742/1706	1.0 (Ref)	
	AT	43/138	0.7 (0.5–1.0)		46/163	0.7 (0.5–0.9)		89/301	0.7 (0.5–0.9)	
	TT	1/4	0.7 (0.1–6.2)	0.17^D^	1/7	0.4 (0.0–3.3)	0.06^D^	2/11	0.5 (0.1–2.1)	0.01^D^
	Per allele		0.7 (0.5–1.0)	0.06^E^		0.7 (0.5–0.9)	0.01^E^		0.7 (0.5–0.9)	0.002^E^
rs516115										
	AA	224/462	1.0 (Ref)		229/543	1.0 (Ref)		453/1005	1.0 (Ref)	
	AG	148/390	0.8 (0.6–1.0)		164/442	0.9 (0.7–1.1)		312/832	0.8 (0.7–1.0)	
	GG	23/67	0.7 (0.4–1.2)	0.14^D^	37/109	0.8 (0.5–1.2)	0.43^D^	60/176	0.8 (0.6–1.1)	0.07^D^
	Per allele		0.8 (0.7–1.0)	0.06^E^		0.9 (0.8–1.1)	0.20^E^		0.9 (0.8–1.0)	0.03^E^
rs741212										
	AA	313/699	1.0 (Ref)		325/830	1.0 (Ref)		638/1529	1.0 (Ref)	
	AG	81/210	0.9 (0.6–1.2)		103/247	1.1 (0.8–1.4)		184/457	1.0 (0.8–1.2)	
	GG	3/11	0.7 (0.2–2.4)	0.53^D^	8/19	1.1 (0.5–2.7)	0.88^D^	11/30	1.0 (0.5–1.9)	0.93^D^
	Per allele		0.9 (0.7–1.1)	0.26^E^		1.1 (0.8–1.3)	0.61^E^		1.0 (0.8–1.2)	0.71^E^
*LUM*										
rs17018765^B^										
	AA	354/776	1.0 (Ref)		395/939	1.0 (Ref)		749/1715	1.0 (Ref)	
	GA	42/141	0.7 (0.5–1.0)		40/152	0.6 (0.4–0.9)		82/293	0.7 (0.5–0.8)	
	GG	1/3	0.9 (0.1–8.6)	0.12^D^	1/7	0.4 (0.0–3.3)	0.03^D^	2/10	0.5 (0.1–2.4)	0.005^D^
	Per allele		0.7 (0.5–1.0)	0.06^E^		0.6 (0.4–0.9)	0.008^E^		0.6 (0.5–0.8)	0.001^E^

A Adjusted for region of residence (Minnesota, Iowa, Wisconsin, Illinois, North Dakota, South Dakota and North Carolina) for MAY and NCO studies; and adjusted for site (MAY, NCO and AUS) for the combined analysis. Sample is 1,317 Caucasian subjects in the discovery set and 1,534 Caucasian subjects in replication set 1.

B Imputed in replication set 1 (AUS).

C Imputed in discovery set (MAY and NCO).

D P-value for two degrees of freedom test.

E P-value for the ordinal model.

In replication set 1, decreased risk associations were found under both co-dominant and ordinal models at *DCN* rs3138165 (P_trend_ = 0.009), *DCN* rs13312816 (P_trend_ = 0.01) and *LUM* rs17018765 (P_trend_ = 0.008) but not at *DCN* rs516115 (P_trend_ = 0.20) or *DCN* rs741212 (P_trend_ = 0.61) (Table 2). Imputed genotypes tended to assume similar risk associations as typed SNPs, likely from high LD among these variants (Figure S1). The squared correlation between imputed and true genotypes was 0.73 for *DCN* rs3138165, 0.73 for *DCN* rs13312816, and 0.68 for *LUM* rs17018765.

The discovery set and replication set 1 were combined in the absence of OR heterogeneity (P_heterogeneity_≥0.48) to increase statistical power. The decreased risk associations remained evident at *DCN* rs3138165 (OR = 0.7; P_trend_ = 0.002), *DCN* rs13312816 (OR = 0.7; P_trend_ = 0.002), *DCN* rs516115 (OR = 0.9; P_trend_ = 0.03) and *LUM* rs17018765 (OR = 0.6; P_trend_ = 0.001) under both co-dominant and ordinal models (Table 2).

**Figure 1:**
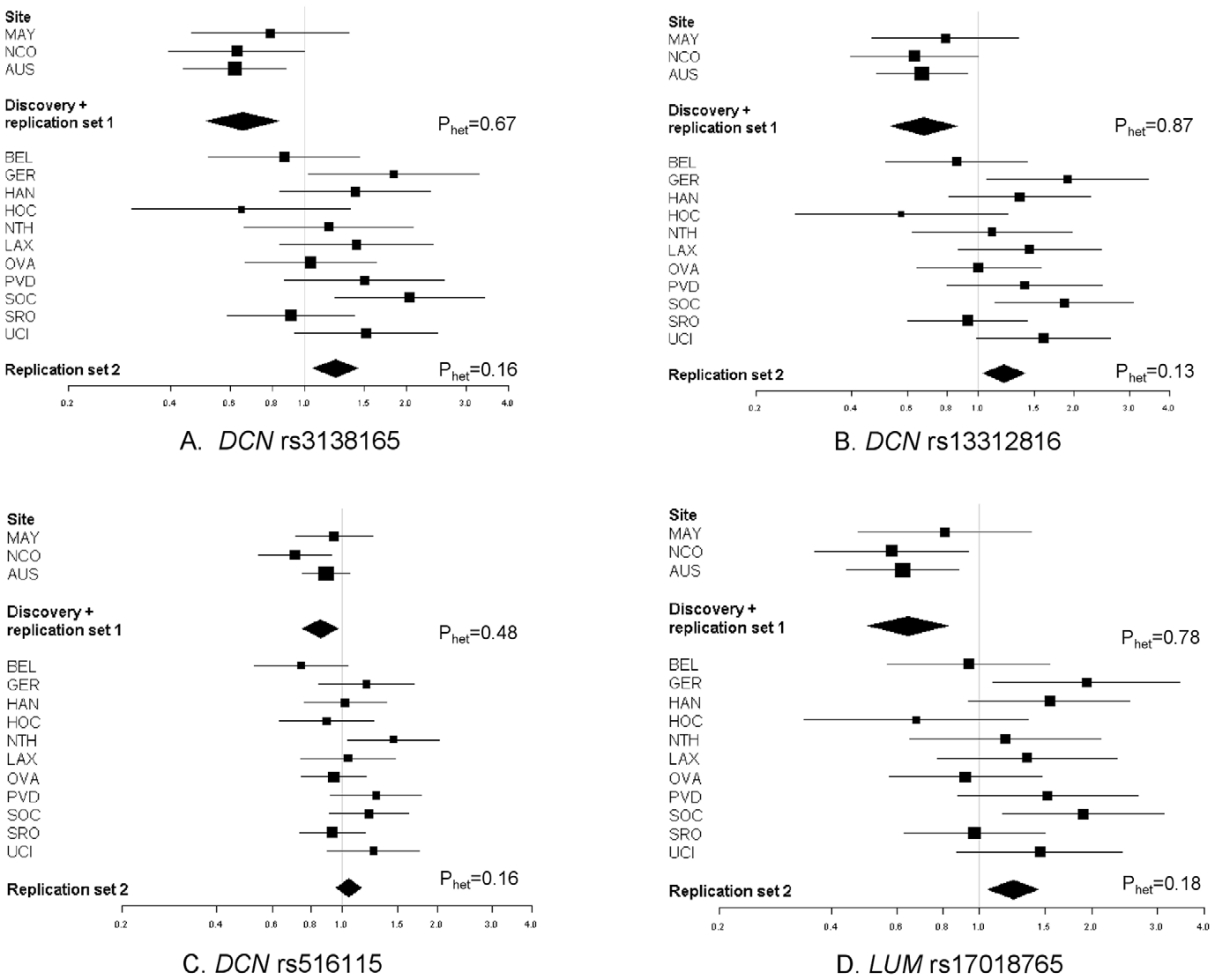
Forest plots for *DCN* and *LUM* SNPs and serous epithelial ovarian cancer. Associations represent ORs (95% CI) for the individual study (squares) and study-adjusted pooled (diamonds) estimates. Models are ordinal genetic risk model. HAN-HJO and HAN-HMO were combined for presentation.

Associations at these four SNPs were tested further in the OCAC replication set 2, and *DCN* rs3138165 and rs13312816, and *LUM* rs17018765 showed statistically significant increased risks (Figure 1, Table S4). Within the OCAC replication set 2, the ORs were statistically homogeneous (P_heterogeneity_≥0.13), but not when combined with the discovery set and replication set 1 (P_heterogeneity_ = 0.001 to 0.03). Heterogeneous ORs were not due to errors in allele coding.

**Figure 2:**
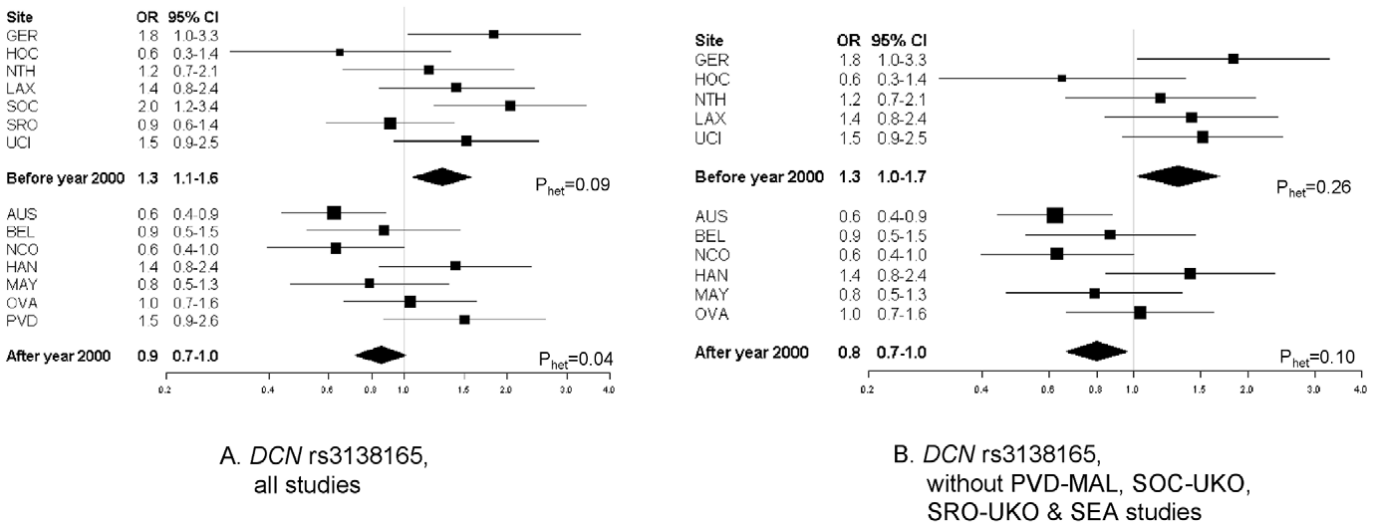
Sensitivity analysis for *DCN* rs3138165 and serous epithelial ovarian cancer stratified by case recruitment period. Associations represent ORs (95% CI) for individual study (squares) and study-adjusted pooled (diamonds) estimates. Models are ordinal genetic risk models. HAN-HJO and HAN-HMO were combined for presentation. P_het_ refers to P value for heterogeneity in odds ratios among studies.

Information on age at diagnosis (or age at interview for controls) and on years of study recruitment was available for all studies and was used to test for SNP interactions in post-hoc analyses in an effort to explain the OR heterogeneity across studies (Table 3). For example, the interaction between *DCN* rs3138165 and age group was suggestive (P_interaction with age_ = 0.04) and per-minor allele associations were highest among women age<40 years (OR = 2.1; P = 0.01; 104 cases) and lowest among women ≥70 years (OR = 0.8; P = 0.24; 510 cases). Results were similar for the three other SNPs (P_interaction with age_ = 0.07 to 0.08; data not shown). Associations at *DCN* rs3138165, stratified by period of recruitment, are shown in Figure 2A, and in Figures S2A, S3A and S4A for the three other SNPs. The per-minor allele summary OR was 1.3 (P = 0.01; 1,007 cases) for studies with a median year of recruitment before 2000, and 0.9 (P = 0.07; 1,494 cases) after 2000 (all SNPs: P_interaction with period_ = 0.002 to 0.01). Because of the modifying effects of period of recruitment and potentially of age, we performed a sensitivity analysis by excluding those case-only studies that were not matched on age and year of recruitment to controls from other studies. As shown in Figure 2B for *DCN* rs3138165, and Figures S2B, S3B and S4Bfor the three other SNPs, the per-minor allele summary ORs were relatively unchanged for studies with a median year of recruitment before 2000 (OR = 1.3; P = 0.03; 612 cases), and after 2000 (OR = 0.8; P = 0.01; 1,340 cases) (all SNPs: P_interaction with period_ = 0.002 to 0.003). However, there was a 20% change in the coefficient for the interaction term. Likewise, changes in the coefficient for the interaction term were 19% for *DCN* rs13312816, 38% for *DCN* rs516115 and 16% for *LUM* rs17018765, consistent with the definition of an important change-in-estimate effect [16]. This is particularly true for *DCN* rs516115, which showed no statistically significant association in the OCAC replication set 2.

**Table 3:** Per-allele odds ratios (OR) and 95% confidence intervals (CI) at *DCN* rs3138165 with serous epithelial ovarian cancer across strata of risk factors.

	Cases/Controls	OR (95% CI)	P value	P interaction
Age at diagnosis/interview, yrs^A^				0.04
<40	104/938	2.1 (1.2–3.5)	0.01	
40–49	406/1,205	1.2 (0.9–1.7)	0.18	
50–59	723/1,652	1.0 (0.8–1.3)	0.92	
60–69	753/1,643	1.0 (0.8–1.3)	0.97	
≥70	510/811	0.8 (0.6–1.1)	0.24	
Missing	5/18			
Diagnosis year, continuous^B^	1,044/2,469	–	–	0.07
Missing	7/62			
Diagnosis year, categories^B^				0.09
1993–1995	58/72	1.5 (0.6–3.9)	0.40	
1996–1999	90/290	1.6 (0.8–3.1)	0.15	
2000–2003	465/1,130	0.9 (0.7–1.2)	0.55	
2004–2006	431/977	0.6 (0.4–0.9)	0.01	
Missing	7/62			
Diagnosis year, binary^B^				0.05
1993–1999	148/362	1.5 (0.9–2.5)	0.14	
2000–2006	896/2,107	0.8 (0.6–0.9)	0.02	
Missing	7/62			
Oral contraceptive use^B^				0.31
Ever	604/1,770	0.7 (0.6–1.0)	0.03	
Never	423/728	1.0 (0.7–1.4)	0.84	
Don't know or missing	24/33			
Parity, n^B^				0.16
Nulliparous	98/191	1.2 (0.6–2.2)	0.62	
1–2	376/929	1.0 (0.7–1.4)	0.88	
≥3	573/1,402	0.7 (0.5–0.9)	0.01	
Don't know or missing	4/9			
BMI, kg/m^2^ B				0.12
15–22.9	272/674	0.6 (0.4–1.0)	0.03	
23–25.9	271/656	0.8 (0.6–1.2)	0.35	
26–28.9	198/470	0.8 (0.5–1.3)	0.43	
29–34.5	189/477	0.8 (0.5–1.3)	0.49	
35–49.9	84/194	1.3 (0.7–2.6)	0.43	
<15 or ≥50	37/60			
Menopausal status^B^				0.41
Pre- or peri-menopausal	214/758	1.1 (0.7–1.6)	0.63	
Post-menopausal	798/1,735	0.8 (0.6–1.0)	0.07	
Don't know or missing	39/38			
Age at menarche, yrs^B^				0.62
8–10	34/59	0.7 (0.2–2.2)	0.57	
11	163/359	1.0 (0.6–1.8)	0.96	
12	223/579	0.6 (0.4–1.0)	0.05	
13	255/686	0.8 (0.5–1.2)	0.30	
≥14 and ≤21	312/784	1.0 (0.7–1.4)	0.95	
<8 or >21	64/64	1.0 (0.3–3.7)	0.97	
Family history^B^^,^ C				0.24
No	313/811	0.6 (0.4–0.9)	0.02	
Yes	88/168	0.7 (0.3–1.5)	0.35	
No sisters/daughters	187/386	1.0 (0.6–1.7)	0.86	
Don't know or missing	463/1,166	1.0 (0.7–1.3)	0.86	

A Among 18 participating studies.

B Among AUS, GER, MAY, NCO and UCI studies only.

C Breast or ovarian cancer in mother, sisters or daughters.

Five studies also had detailed information on covariates (Table 3). Interactions are presented between *DCN* rs3138165 and diagnosis year (P_interaction for continuous years_ = 0.07 and P_interaction for binary variable_ = 0.05), OC use (P_interaction_ = 0.31), parity (P_interaction_ = 0.16), BMI (P_interaction_ = 0.12), menopausal status (P_interaction_ = 0.41), age at menarche (P_interaction_ = 0.62) and family history (P_interaction_ = 0.24). Per-minor allele associations by diagnosis year mimicked those of recruitment period (diagnoses prior to year 2000: OR = 1.5; P = 0.14; 148 cases and diagnoses after year 2000: OR = 0.8; P = 0.02; 896 cases). Although the interactions were not statistically significant, per-minor allele associations at *DCN* rs3138165 were associated with lower risk among women who ever took OC hormones (OR = 0.7; P = 0.03; 604 cases), among women with ≥3 full-term births (OR = 0.7; P = 0.01; 573 cases), and among lean women (OR = 0.6; P = 0.03; 272 cases). Associations were similar for the three other SNPs (data not shown). Diagnosis year (1993–1999 vs 2000–2006) was associated with OC use (P<0.0001), parity (P<0.0001), BMI (P<0.0001), menopausal status (P<0.0001), age at menarche (P<0.0001) and family history (P<0.0001), but not with age group (P = 0.32) or *DCN* rs3138165 (P = 0.54), in unadjusted models. In a model fitting all covariates including *DCN* rs3138165 and site, only site (P<0.0001) and age at menarche (P = 0.02) were significantly associated with diagnosis year (data not shown).

## Discussion

We could not confirm the associations of SNPs in two stromal genes, *DCN* and *LUM*, with the risk of serous epithelial ovarian cancer, the most common histological type of ovarian cancer, using a multi-stage replication approach within the OCAC. We found decreased risks at four SNPs in the discovery set and replication set 1, and increased risks in a larger sample of cases in the OCAC replication set 2. The heterogeneity in associations across studies was statistically significant and was explained, in part, by the period of case recruitment, with the four SNPs imparting up to a 30% increased risk for diagnoses before the year 2000 and up to a 20% decreased risk after the year 2000. Weaker interactions were seen with age at diagnosis and with diagnosis year in post-hoc analyses. Non-statistically significant modifying effects of OC use, parity and BMI were also observed.

Age-adjusted incidence rates of epithelial ovarian cancer have been trending lower in most of North America and Europe since the 1990s [17], [18], and since our gene pool is not changing over such a short period, we speculated that our results reflect changes in the environment. As expected, there was no statistically significant association of diagnosis year with *DCN* rs3138165, although there were significant associations with several of the covariates and one of these, age at menarche, remained statistically significant with diagnosis year in the multivariable-adjusted model. Our findings may suggest that temporal changes in risk factors are modifiers of inherited susceptibility in *DCN* and *LUM*. However, we cannot exclude the role of unmeasured factors that are related to diagnosis year or to study site, or that our findings are due to chance. Several of the 15 studies in replication set 2 are new to OCAC, and epidemiological variables for subjects have not yet been submitted to the central Data Coordinating Center. We were, therefore, under-powered to evaluate gene-environment interactions and can only speculate that age, increasing obesity [19], changing trends in OC hormone preparation and use [20], [21], or increasing age at pregnancy/decreasing parity [22] may be obvious candidates for future testing of temporal changes that may modify risk associations of these SNPs. Each of these hormonally-related factors is associated with ovarian cancer [23]–[26]. Studies examining the response of normal ovarian epithelium to hormonal factors reported that macaque primates receiving progestin alone had higher frequencies of apoptotic ovarian epithelial cells compared to control animals or those receiving estrogen alone [27]. Furthermore, the protective effect of parity may be from exposure to pregnancy hormones such as progesterone that have been speculated to clear the ovarian epithelium of precancerous cells [28]. In the macaque study, the increase in apoptotic cells correlated highly with a shift in expression from TGF-β1 isoform to TGF-β2 and -β3 isoforms in the ovarian surface epithelium [29]. TGF-β isoforms appear to be regulated by a variety of steroid hormones in a tissue-specific manner (reviewed in [29]). In contrast, ovarian carcinomas are frequently resistant to TGF-β-mediated growth inhibition [30], [31] and express higher levels of TGF-β1 and TGF-β3, and lower levels of TGF-β2, than normal human ovarian specimens [30], the significance of which is unclear.

Decorin and lumican have multiple biological roles including control of cell proliferation [32]. Interestingly, decorin belongs to the family of secretory glycoproteins known as latent TGF-β-binding proteins (LTBPs) that sequesters the pro-hormone or latent form of TGF-β and prevents it from interacting with its signaling receptors, TβRI and TβRII [15], [33]. LTBPs may facilitate the secretion, storage or activation of latent TGF-βs and serve as a reservoir for concentrated delivery of TGF-βs to receptors [15]. TGF-βs [33] and decorin [34] have been implicated as potent tumor suppressors; however, the diverse array of cellular processes regulated by TGF-βs seems to depend on the microenvironment: for example, promoting apoptosis and inhibiting epithelial growth in normal cells and promoting proliferation and angiogenesis in various cancer models [15], [35]. The link between progesterone, TGF-βs and decorin is particularly intriguing within the context of our SNP-environment associations. However, this investigation was not designed to examine SNP-environment interactions and the power to detect significant effect modification with the available sample size was low.

We also compared our results to unpublished findings from two recent genome wide association (GWA) studies of ovarian cancer, but there was no clear support for associations at the SNPs. For example, the four SNPs were not associated statistically with serous epithelial ovarian cancer (ORs = 0.93–0.96; P = 0.28–0.76) in phase 1 of a GWA study comprising 870 Caucasian cases from the United Kingdom [36]. Among 3,248 serous epithelial ovarian cancers in a GWA study of Caucasians from the United States, of which approximately 12% were composed of the 397 cases in our discovery dataset, we observed modest associations at the *DCN* SNPs (ORs = 0.82–0.87; P = 0.02–0.06) and at the *LUM* SNP (OR = 1.20; 95% CI = 0.97–1.48; P = 0.09) (unpublished findings). The discrepancies in results likely reflect similar challenges in interpretation as the OCAC results and underscore the importance of understanding the distribution of individual-level environmental exposures in genetic studies [37].

The strengths of this study include the multi-stage replication strategy, representing 2,501 total serous epithelial ovarian cancer cases. To reduce the impact of population stratification, our analyses were restricted to known or presumed Caucasians. Although one study (SRO) consisted of mostly Caucasians, our results were unchanged when this study was excluded in sensitivity analyses. The characteristics of the samples from the discovery set and replication set 1 were similarly distributed, as was the period of recruitment, thus reducing the impact of effect modification on the SNP-disease associations in these three studies. By restricting the samples to serous epithelial ovarian cancers, we reduced etiologic heterogeneity that may exist among different histological types of ovarian cancer [38]. Finally, we used statistical techniques to impute untyped SNPs as an efficient approach to include these SNPs in a combined analysis of samples from the discovery set and replication set 1.

The major limitation of this investigation is the absence of epidemiological information for most of the OCAC studies included in this report. Thus, our findings in the post-hoc analyses, while intriguing, require a tempered interpretation. Although MAFs of SNPs were generally similar across OCAC studies, occasionally a 1.5 to 2-fold difference was observed, which might suggest population structure influences on associations. Furthermore, we genotyped tagSNPs, which are likely only proxies for the putative causal SNP(s).

In summary, our multi-stage replication investigation suggests that SNPs in *DCN* and *LUM* are not associated with serous epithelial ovarian cancer. Verification of possible effect modification by age and other unconfirmed temporal effects is underway in an OCAC investigation of 10,000 cases and 10,000 controls.

## Materials and Methods

### Ethics statement

Participants in all the studies provided written informed consent and each site's institutional review board approved the study protocol (Text S1), including Ethics Committees of the Queensland Institute of Medical Research and Peter MacCallum Cancer Centre (AUS); the local Ethical Committee (Commissie Medische Ethiek UZ Leuven, Belgium) (BEL); and Ethics Committee of the University of Heidelberg (GER).

### Discovery set

The discovery set consisted of a combination of two individual studies of epithelial ovarian cancer from MAY and NCO in the United States. Details of the study protocols have been published previously [39]. Briefly, participants included Caucasians and African-Americans enrolled between June 1999 and March 2006 (see Table S5 for detailed study descriptions). In both studies, cases were newly diagnosed, histologically-confirmed, either borderline or invasive, and enrolled within one year of diagnosis. Controls had at least one intact ovary, no history of ovarian cancer and were frequency matched to cases on age (5-yr age categories), race and state of residence.

### Replication set 1

The Australian Ovarian Cancer Study recruited cases diagnosed between January 2002 and June 2006 from surgical treatment centers and cancer registries throughout Australia [40]; recruitment through the New South Wales and Victorian Cancer Registries was conducted under the Australian Cancer Study [41] (together, they form the AUS study). Controls were population-based and were randomly selected from the Australian electoral roll and frequency matched to cases on age and state of residence (Table S5).

### Ovarian Cancer Association Consortium (OCAC) replication set 2

Fifteen studies from Belgium (BEL), Canada (OVA), Denmark (MAL, PVD), Finland (HOC), Germany (GER, HAN-HJO, HAN-HMO), Netherlands (NTH), the United Kingdom (SEA, SOC, SRO, UKO) and the United States (LAX, UCI), comprising 4,536 primary epithelial ovarian cancer cases and 4,622 controls for whom genotype data were available, were used as a second replication set (Table S5). Most were case-control studies, although some of these studies (PVD, SOC, SRO and LAX) consisted of cases-only and were matched by region within country to unique controls from other OCAC studies. Thus, SOC cases were matched to UKO controls, SRO cases to UKO and SEA controls, LAX cases to UCI controls and PVD cases to MAL controls, resulting in 12 matched studies for analysis.

Information on established risk factors (reproductive history, family history of cancer, medical history, and lifestyle habits) including diagnosis year was collected in the discovery set and replication set 1 and was available for two replication set 2 studies (GER, UCI).

### SNP selection, genotyping and quality control (QC)

#### Discovery set

Tag single nucleotide polymorphisms (SNPs) were chosen from unrelated Caucasian samples within HapMap Consortium's release 22 [42] as previously described [43], and also for their predicted likelihood of successful genotyping using the Illumina Golden Gate Assay™. We identified six tagSNPs from among 22 *DCN* SNPs and seven tagSNPs from among 16 *LUM* SNPs with minor allele frequency (MAF)≥0.05 and pairwise linkage disequilibrium (LD) of r^2^≥0.8. One tagSNP in each gene was predicted to assay poorly (design score = 0), was a singleton in its bin and could not be replaced. This left five tagSNPs in *DCN* (rs3138165, rs516115, rs10492230, rs741212, and rs3138268) and six tagSNPs in *LUM* (rs17714469, rs1920790, rs2268578, rs10859110, rs10745553, and rs17018765), including five putative functional SNPs, for genotyping. The SNPs were located within, and 5 kb upstream and downstream of, each gene region. The two genes comprise a contiguous segment on chromosome 12 of approximately 80 kb.

The 11 tagSNPs were genotyped as part of a larger investigation of 1,152 SNPs in a variety of pathways using the Illumina GoldenGate™ assay and Illumina BeadStudio software [44]. Genotyping was attempted on 897 DNA samples from MAY and 1,279 samples from NCO (total = 2,176 including 129 duplicate samples) and 65 laboratory controls. Case status and duplicate samples were blinded to laboratory personnel who performed the genotyping. Of these samples, we excluded 44 with call rates <90% and Illumina QC (GenCall) scores<0.25, and 22 ineligible or mislabelled samples, resulting in 1,981 unique samples that were successfully genotyped. The sample call rate was 99.74% and the concordance for duplicate samples was 99.99%. *DCN* rs3138268, a nonsynonymous SNP, was monomorphic and was excluded from further analyses. The remaining 10 tagSNPs were genotyped successfully.

#### Replication set 1

Three tagSNPs in *DCN* (rs13312816, rs516115, and rs741212) were genotyped as part of a larger assay of 1,536 SNPs in AUS (*LUM* SNPs were not genotyped). Genotyping was attempted on 1,674 samples using the Illumina GoldenGate™ assay and Illumina BeadStudio software [44]. One non-template control and two DNA samples per 96-well plate were blindly duplicated (n = 18). Samples with call rates <95% and SNPs with call rates <98% were excluded. SNPs with GenTrain scores (a metric of genotype clustering)<0.5 were manually checked and adjusted according to Illumina guidelines. Greater than 97% of SNPs passed this initial QC and >84% of all SNPs passed all QC criteria, resulting in 550 cases and 1,101 controls (93% Caucasians) with genotype data on 1,292 SNPs, including the three tagSNPs in *DCN* included in this analysis.

#### Imputation

SNPs genotyped in the discovery set were not necessarily the same SNPs genotyped in replication set 1 and vice versa (e.g., only *DCN* rs516115 and *DCN* rs741212 were genotyped in both datasets). Genotypes at SNPs showing significant associations with ovarian cancer in either dataset were imputed so that datasets could be combined for analysis. Thus, we imputed *DCN* rs13312816 in the discovery set and *DCN* rs3138165 and *LUM* rs17018765 in replication set 1 using the MACH software [45]. Briefly, genotype data from the discovery set and replication set 1 were combined with the phase II HapMap data for Caucasian samples and the unobserved genotypes were then inferred probabilistically using a hidden Markov model [45].

#### OCAC replication set 2

We genotyped four SNPs (*DCN* SNPs rs3138165, rs13312816 and rs516115 and *LUM* rs17018765) showing significant associations in the discovery set and replication set 1 in the 12 matched OCAC studies using the Fluidigm EP1 system (Fluidigm, San Francisco, CA) at a central laboratory. Genotyping was performed on 96.96 dynamic arrays in a run of 96 SNPs using inventoried and Custom Assay-by-Design TaqMan probes (Applied Biosystems, Foster City, CA). Genotyping used 10 ng DNA following the manufacturer's conditions using the pre-amplification protocol. Analysis was performed using Genotyping SNP Analysis software. Samples with call rates <80% were excluded immediately. The following criteria were used as measures of acceptable genotyping for each SNP and each matched study set: (i) ≥2% sample duplicates included, (ii) concordance for duplicate samples ≥96% and overall concordance for duplicate samples across all SNPs ≥98%, (iii) pass rate per plate of >90%, (iv) <25% overall failed plates, (v) overall SNP call rate by study ≥95%, and (vi) a difference in call rate between cases and controls of <5%. Studies failing one of these criteria were excluded for that particular SNP, resulting in 8,886 unique samples (4,419 cases and 4,467 controls) that were successfully genotyped. Excellent concordance (100%) in genotype calls was found between study samples and those of 95 HapMap genotyped DNAs (Coriell, Camden, NJ, USA).

For all studies, genotyping quality was further assessed using tests for Hardy-Weinberg equilibrium (HWE). SNPs with significant deviations from HWE in Caucasian controls (0.001<P<0.05) were assessed and excluded if the clustering was suboptimal. SNPs with HWE P<0.001 were excluded from analysis.

### Statistical Analysis

We restricted analyses to subjects who were self-reported or presumed Caucasian and cases with invasive epithelial ovarian cancer of serous histology, resulting in a final sample size of 1,317 participants in the discovery set (397 cases and 920 controls), 1,534 participants in replication set 1 (436 cases and 1,098 controls) and 5,917 participants in OCAC replication set 2 (1,668 cases and 4,249 controls). Genotypes were used to estimate allele frequencies and pair-wise LD between SNPs was estimated with r^2^ values using Haploview version 4.1 [46].

We estimated odds ratios (OR) and 95% confidence intervals (CI) at each SNP using unconditional logistic regression under both co-dominant and ordinal genetic models. In the discovery set only, we also estimated haplotype frequencies for each gene and tested the global statistical significance (P<0.05) for haplotype association [47]. Individual haplotype associations evaluated the risk of serous epithelial ovarian cancer compared to all other haplotypes combined.

**Figure 3:**
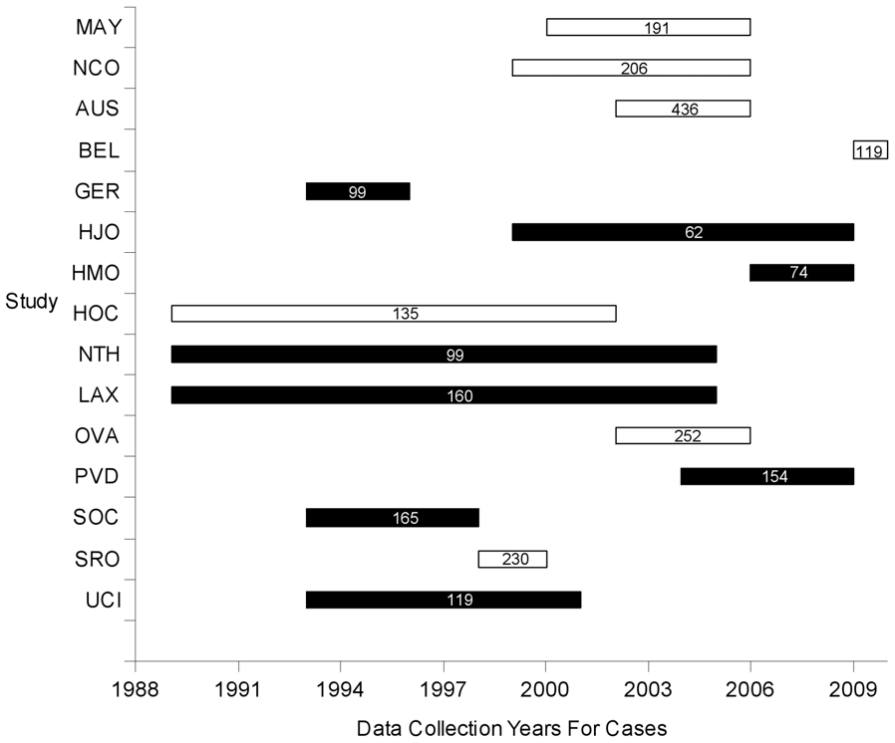
Recruitment years for cases in each study. White bars denote studies showing no association or inverse associations between *DCN* or *LUM* SNPs with serous epithelial ovarian cancer, whereas black bars denote studies showing positive associations. Numbers in bars represent number of cases used in analyses.

Prior to combining data, statistical tests of heterogeneity in the ORs between studies were evaluated. Where heterogeneity existed, statistical significance of interaction in post-hoc analyses was assessed with the Wald test in models that included a product term for the ordinal coding of genotype and categories of age or period of recruitment (before the year 2000 or after the year 2000 based on the median year of the recruitment duration for each study, Figure 3) while adjusting for study. Among the five studies (AUS, GER, MAY, NCO and UCI) with detailed information on covariates, we also examined SNP interactions with diagnosis year, oral contraceptive (OC) use, parity, body mass index (BMI), menopausal status, age at menarche and family history of breast or ovarian cancer in first degree relatives. Missing observations were represented as a separate category within each variable. Associations representing the ordinal genetic model at each SNP were then stratified by the covariate. All models were adjusted for region of residence (discovery set only) or study. Additional adjustment for age categories did not alter associations, so models are presented without age.

All statistical tests were two-sided with an alpha level<0.05 considered statistically significant, and were implemented with SAS (SAS Institute, NC).

## Supporting Information

Figure S1 **Linkage disequilibrium blocks for tagSNPs in** ***DCN*** **and** ***LUM*** **.** Analysis is based on total number of controls from the discovery set and replication set 1. The two genes comprise a contiguous segment on chromosome 12 of approximately 80 kb. Numbers in the squares on the LD block indicate the correlation (r^2^) between SNPs. * indicates SNPs that were significantly associated with serous ovarian cancer.
(TIF)

Figure S2 **Sensitivity analysis for** ***DCN*** **rs13312816 and serous epithelial ovarian cancer stratified by case recruitment period.** Forest plots represent associations represent ORs (95% CI) for individual study (squares) and study-adjusted pooled (diamonds) estimates. Models are ordinal genetic risk models. HAN-HJO and HAN-HMO were combined for presentation. P_het_ refers to P value for heterogeneity in odds ratios among studies.
(TIF)

Figure S3 **Sensitivity analysis for** ***DCN*** **rs516115 and serous epithelial ovarian cancer stratified by case recruitment period.** Forest plots represent associations represent ORs (95% CI) for individual study (squares) and study-adjusted pooled (diamonds) estimates. Models are ordinal genetic risk models. HAN-HJO and HAN-HMO were combined for presentation. P_het_ refers to P value for heterogeneity in odds ratios among studies.
(TIF)

Figure S4 **Sensitivity analysis for** ***LUM*** **rs17018765 and serous epithelial ovarian cancer stratified by case recruitment period.** Forest plots represent associations represent ORs (95% CI) for individual study (squares) and study-adjusted pooled (diamonds) estimates. Models are ordinal genetic risk models. HAN-HJO and HAN-HMO were combined for presentation. P_het_ refers to P value for heterogeneity in odds ratios among studies.
(TIF)

Table S1 SNP and location, HWE test P-value and MAF for variants in *DCN* and *LUM* in 920 controls in the discovery set and 1,098 controls in replication set 1.
(DOC)

Table S2 Odds ratios (OR) and 95% confidence intervals (CI) for the association between genetic polymorphisms in *DCN* and *LUM* and serous epithelial ovarian cancer risk among 1,317 Caucasian subjects in the discovery set.
(DOC)

Table S3 Haplotype analysis of decorin and lumican genes and invasive serous epithelial ovarian cancer risk among 1,317 Caucasian subjects in the discovery set.
(DOC)

Table S4 Genotype counts, MAF and HWE statistics and associations between genotypes and risk of ovarian carcinoma for *DCN* and *LUM* SNPs among Caucasian subjects in OCAC replication set 2.
(DOC)

Table S5 Overview of 18 OCAC studies with serous epithelial ovarian cancer cases and controls.
(DOC)

Text S1 Ethics statement.
(DOC)

## References

[1] Jemal A, Siegel R, Ward E, Hao Y, Xu J (2008). Cancer statistics, 2008.. CA Cancer J Clin.

[2] Piver MS, Baker TR, Piedmonte M, Sandecki AM (1991). Epidemiology and etiology of ovarian cancer.. Semin Oncol.

[3] Ronnov-Jessen L, Petersen OW, Bissell MJ (1996). Cellular changes involved in conversion of normal to malignant
breast: importance of the stromal reaction.. Physiol Rev.

[4] Bishop JM (1995). Cancer: the rise of the genetic paradigm.. Genes Dev.

[5] Barcellos-Hoff MH, Ravani SA (2000). Irradiated mammary gland stroma promotes the expression of
tumorigenic potential by unirradiated epithelial cells.. Cancer Res.

[6] Barcellos-Hoff MH (1993). Radiation-induced transforming growth factor beta and subsequent
extracellular matrix reorganization in murine mammary gland.. Cancer Res.

[7] Barcellos-Hoff MH, Derynck R, Tsang ML, Weatherbee JA (1994). Transforming growth factor-beta activation in irradiated murine
mammary gland.. J Clin Invest.

[8] Iozzo RV (1997). The family of the small leucine-rich proteoglycans: key
regulators of matrix assembly and cellular growth.. Crit Rev Biochem Mol Biol.

[9] Koninger J, Giese NA, di Mola FF, Berberat P, Giese T (2004). Overexpressed decorin in pancreatic cancer: potential tumor
growth inhibition and attenuation of chemotherapeutic
action.. Clin Cancer Res.

[10] Leygue E, Snell L, Dotzlaw H, Troup S, Hiller-Hitchcock T (2000). Lumican and decorin are differentially expressed in human breast
carcinoma.. J Pathol.

[11] Shridhar V, Lee J, Pandita A, Iturria S, Avula R (2001). Genetic analysis of early- versus late-stage ovarian
tumors.. Cancer Res.

[12] Nash MA, Deavers MT, Freedman RS (2002). The expression of decorin in human ovarian
tumors.. Clin Cancer Res.

[13] Grisaru D, Hauspy J, Prasad M, Albert M, Murphy KJ (2007). Microarray expression identification of differentially expressed
genes in serous epithelial ovarian cancer compared with bulk normal ovarian
tissue and ovarian surface scrapings.. Oncol Rep.

[14] Grazio D, Pichler I, Fuchsberger C, Zolezzi F, Guarnieri P (2008). Differential gene expression analysis of ovarian cancer in a
population isolate.. Eur J Gynaecol Oncol.

[15] Massague J, Chen YG (2000). Controlling TGF-beta signaling.. Genes Dev.

[16] Greenland S (1989). Modeling and variable selection in epidemiologic
analysis.. Am J Public Health.

[17] National Cancer Institute Surveillance Epidemiology and End
Results Cancer Statistics Review 1975–2007.. http://seer.cancer.gov/csr/1975_2007/browse_csr.php.

[18] Bray F, Loos AH, Tognazzo S, La Vecchia C (2005). Ovarian cancer in Europe: Cross-sectional trends in incidence and
mortality in 28 countries, 1953–2000.. Int J Cancer.

[19] Flegal KM, Carroll MD, Ogden CL, Curtin LR (2010). Prevalence and trends in obesity among US adults,
1999–2008.. JAMA.

[20] Gerstman BB, Gross TP, Kennedy DL, Bennett RC, Tomita DK (1991). Trends in the content and use of oral contraceptives in the
United States, 1964–88.. Am J Public Health.

[21] Gerstman BB, Burke L, Delaney J, McLellan B (1996). Steroidal contraceptive use update, United States,
1989–1994.. Pharmacoepidemiol Drug Saf.

[22] Matthews TJ, Hamilton BE (2009). Delayed childbearing: more women are having their first child
later in life.. NCHS Data Brief.

[23] Reeves GK, Pirie K, Beral V, Green J, Spencer E (2007). Cancer incidence and mortality in relation to body mass index in
the Million Women Study: cohort study.. BMJ.

[24] Beral V, Doll R, Hermon C, Peto R, Reeves G (2008). Ovarian cancer and oral contraceptives: collaborative reanalysis
of data from 45 epidemiological studies including 23,257 women with ovarian
cancer and 87,303 controls.. Lancet.

[25] Whiteman DC, Siskind V, Purdie DM, Green AC (2003). Timing of pregnancy and the risk of epithelial ovarian
cancer.. Cancer Epidemiol Biomarkers Prev.

[26] Risch HA, Marrett LD, Howe GR (1994). Parity, contraception, infertility, and the risk of epithelial
ovarian cancer.. Am J Epidemiol.

[27] Rodriguez GC, Walmer DK, Cline M, Krigman H, Lessey BA (1998). Effect of progestin on the ovarian epithelium of macaques: cancer
prevention through apoptosis?. J Soc Gynecol Investig.

[28] Adami HO, Hsieh CC, Lambe M, Trichopoulos D, Leon D (1994). Parity, age at first childbirth, and risk of ovarian
cancer.. Lancet.

[29] Rodriguez GC, Nagarsheth NP, Lee KL, Bentley RC, Walmer DK (2002). Progestin-induced apoptosis in the Macaque ovarian epithelium:
differential regulation of transforming growth factor-beta.. J Natl Cancer Inst.

[30] Bristow RE, Baldwin RL, Yamada SD, Korc M, Karlan BY (1999). Altered expression of transforming growth factor-beta ligands and
receptors in primary and recurrent ovarian carcinoma.. Cancer.

[31] Yamada SD, Baldwin RL, Karlan BY (1999). Ovarian carcinoma cell cultures are resistant to
TGF-beta1-mediated growth inhibition despite expression of functional
receptors.. Gynecol Oncol.

[32] Zafiropoulos A, Tzanakakis GN (2008). Decorin-mediated effects in cancer cell biology.. Connect Tissue Res.

[33] Shi Y, Massague J (2003). Mechanisms of TGF-beta signaling from cell membrane to the
nucleus.. Cell.

[34] De Luca A, Santra M, Baldi A, Giordano A, Iozzo RV (1996). Decorin-induced growth suppression is associated with
up-regulation of p21, an inhibitor of cyclin-dependent
kinases.. J Biol Chem.

[35] Siegel PM, Massague J (2003). Cytostatic and apoptotic actions of TGF-beta in homeostasis and
cancer.. Nat Rev Cancer.

[36] Song H, Ramus SJ, Tyrer J, Bolton KL, Gentry-Maharaj A (2009). A genome-wide association study identifies a new ovarian cancer
susceptibility locus on 9p22.2.. Nat Genet.

[37] Ioannidis JP, Thomas G, Daly MJ (2009). Validating, augmenting and refining genome-wide association
signals.. Nat Rev Genet.

[38] Kobel M, Kalloger SE, Boyd N, McKinney S, Mehl E (2008). Ovarian carcinoma subtypes are different diseases: implications
for biomarker studies.. PLoS Med.

[39] Sellers TA, Schildkraut JM, Pankratz VS, Vierkant RA, Fredericksen ZS (2005). Estrogen bioactivation, genetic polymorphisms, and ovarian
cancer.. Cancer Epidemiol Biomarkers Prev.

[40] Beesley J, Jordan SJ, Spurdle AB, Song H, Ramus SJ (2007). Association between single-nucleotide polymorphisms in hormone
metabolism and DNA repair genes and epithelial ovarian cancer: results from
two Australian studies and an additional validation set.. Cancer Epidemiol Biomarkers Prev.

[41] Merritt MA, Green AC, Nagle CM, Webb PM (2008). Talcum powder, chronic pelvic inflammation and NSAIDs in relation
to risk of epithelial ovarian cancer.. Int J Cancer.

[42] The International HapMap Consortium (2003). The International HapMap Project.. Nature.

[43] Kelemen LE, Sellers TA, Schildkraut JM, Cunningham JM, Vierkant RA (2008). Genetic variation in the one-carbon transfer pathway and ovarian
cancer risk.. Cancer Res.

[44] Oliphant A, Barker DL, Stuelpnagel JR, Chee MS (2002). BeadArray technology: enabling an accurate, cost-effective
approach to high-throughput genotyping.. Biotechniques.

[45] Li Y, Willer C, Sanna S, Abecasis G (2009). Genotype imputation.. Annu Rev Genomics Hum Genet.

[46] Barrett JC, Fry B, Maller J, Daly MJ (2005). Haploview: analysis and visualization of LD and haplotype
maps.. Bioinformatics.

[47] Scaid DJ (2010). Haplo.stats.. http://mayoresearch.mayo.edu/mayo/research/biostat/schaid.cfm.

