# Supplementary figures and images for: Polymorphisms in Stromal Genes and Susceptibility to Serous Epithelial Ovarian Cancer: A Report from the Ovarian Cancer Association Consortium
Source: PLoS One. 2011 May 27;6(5):e19642. doi: 10.1371/journal.pone.0019642 (PMC3103497; doi:10.1371/journal.pone.0019642)

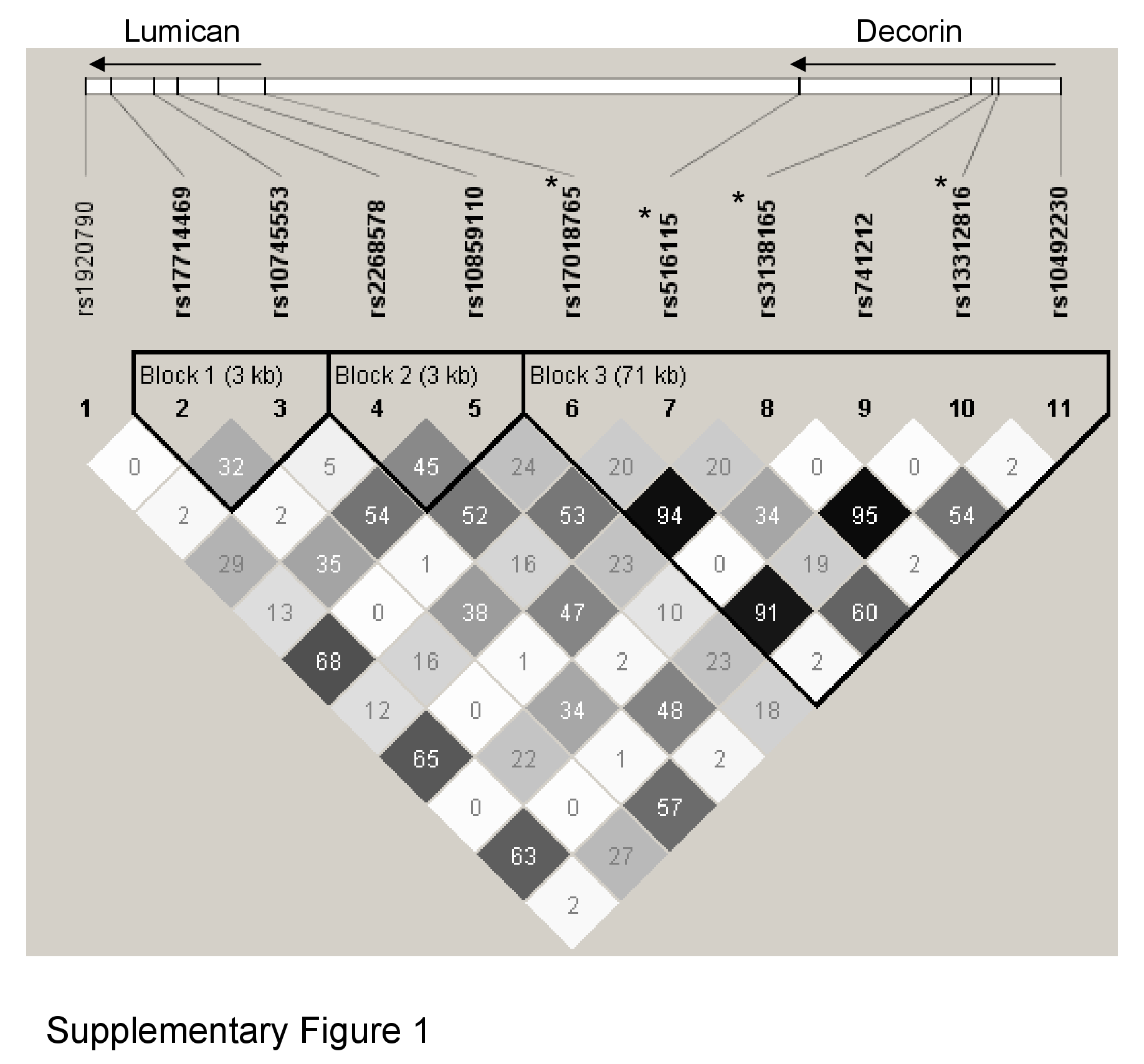

Supplement: Figure S1 — Linkage disequilibrium blocks for tagSNPs in DCN and LUM . Analysis is based on total number of controls from the discovery set and replication set 1. The two genes comprise a contiguous segment on chromosome 12 of approximately 80 kb. Numbers in the squares on the LD block indicate the correlation (r2) between SNPs. * indicates SNPs that were significantly associated with serous ovarian cancer. (TIF) [file pone.0019642.s001.tif]

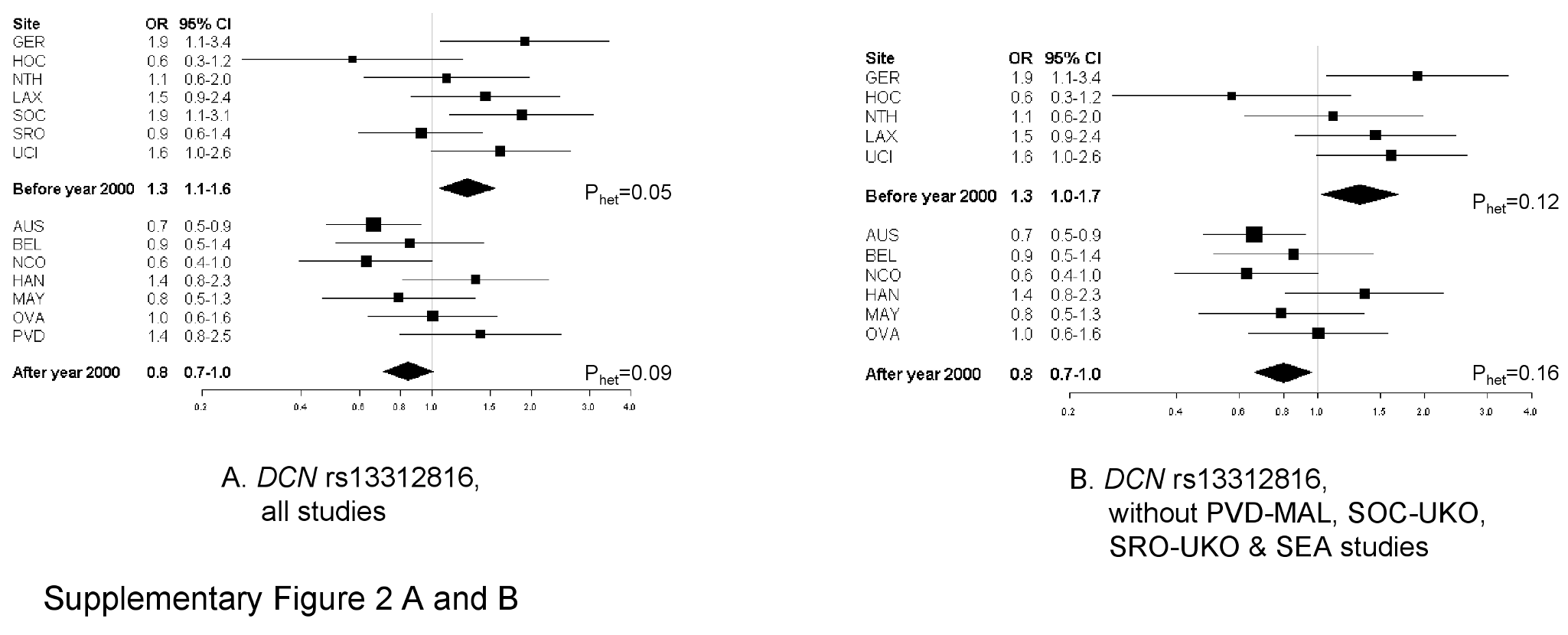

Supplement: Figure S2 — Sensitivity analysis for DCN rs13312816 and serous epithelial ovarian cancer stratified by case recruitment period. Forest plots represent associations represent ORs (95% CI) for individual study (squares) and study-adjusted pooled (diamonds) estimates. Models are ordinal genetic risk models. HAN-HJO and HAN-HMO were combined for presentation. Phet refers to P value for heterogeneity in odds ratios among studies. (TIF) [file pone.0019642.s002.tif]

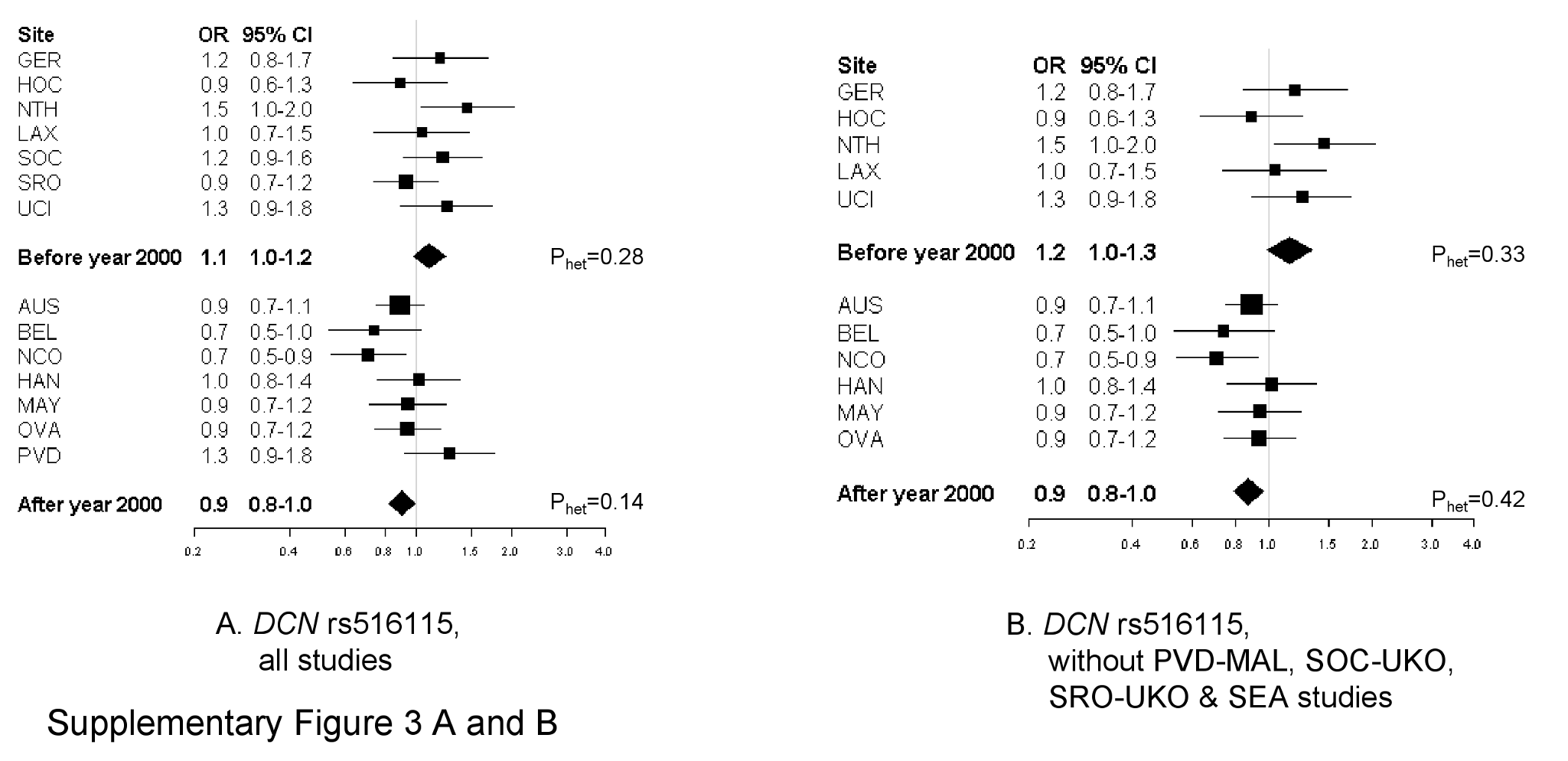

Supplement: Figure S3 — Sensitivity analysis for DCN rs516115 and serous epithelial ovarian cancer stratified by case recruitment period. Forest plots represent associations represent ORs (95% CI) for individual study (squares) and study-adjusted pooled (diamonds) estimates. Models are ordinal genetic risk models. HAN-HJO and HAN-HMO were combined for presentation. Phet refers to P value for heterogeneity in odds ratios among studies. (TIF) [file pone.0019642.s003.tif]

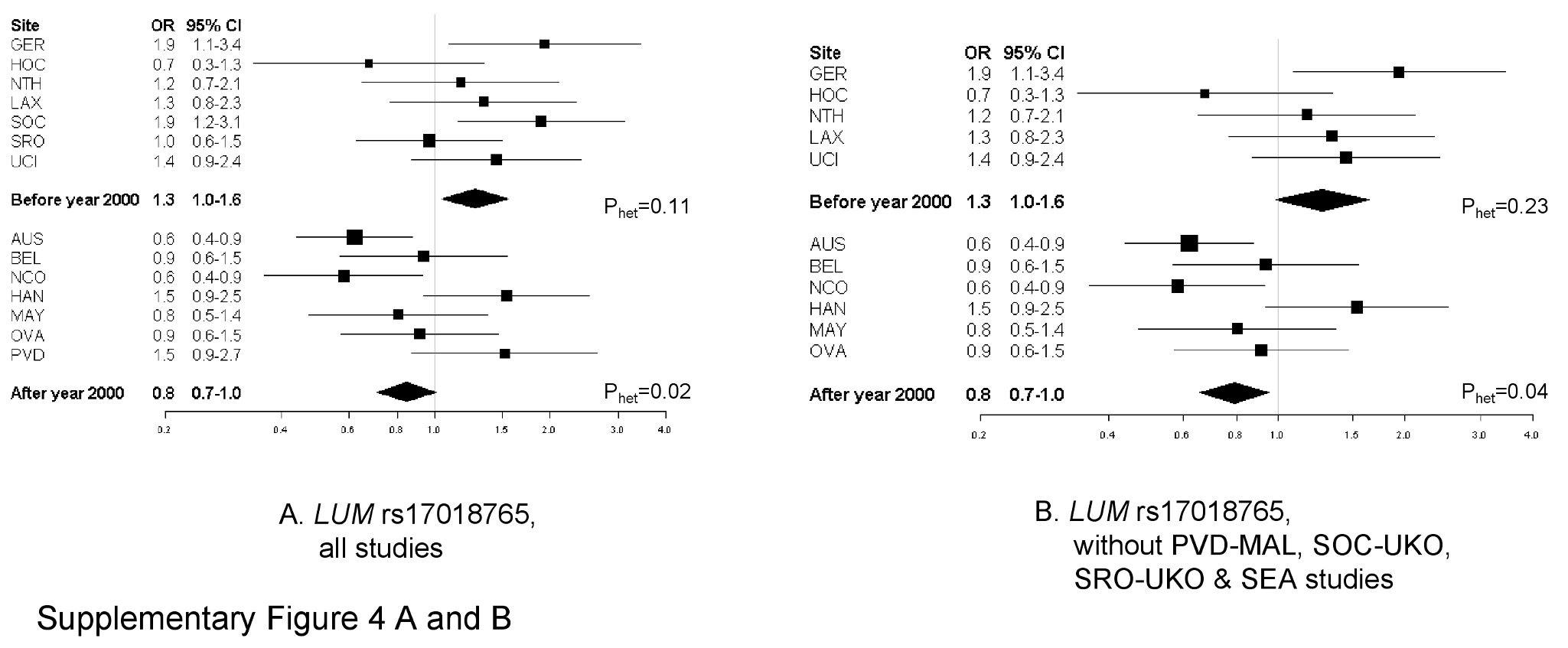

Supplement: Figure S4 — Sensitivity analysis for LUM rs17018765 and serous epithelial ovarian cancer stratified by case recruitment period. Forest plots represent associations represent ORs (95% CI) for individual study (squares) and study-adjusted pooled (diamonds) estimates. Models are ordinal genetic risk models. HAN-HJO and HAN-HMO were combined for presentation. Phet refers to P value for heterogeneity in odds ratios among studies. (TIF) [file pone.0019642.s004.tif]
