# Supplementary material for: Polymorphisms in Stromal Genes and Susceptibility to Serous Epithelial Ovarian Cancer: A Report from the Ovarian Cancer Association Consortium
Source: PLoS One. 2011 May 27;6(5):e19642. doi: 10.1371/journal.pone.0019642 (PMC3103497; doi:10.1371/journal.pone.0019642)
Supplement: Table S1 — SNP and location, HWE test P-value and MAF for variants in DCN and LUM in 920 controls in the discovery set and 1,098 controls in replication set 1. (DOC) [file pone.0019642.s005.doc]

**Table S1. SNP and location, HWE test P-value and MAF for variants in *DCN* and *LUM*** in 920 controls in the discovery set and 1,098 controls in replication set 1

| Gene | Chr position (bp)^A^ | SNP rsID | Major/Minor Allele | Location in gene | MAF^B^ | | HWE^B^ test p-value | |
| --- | --- | --- | --- | --- | --- | --- | --- | --- |
|  |  |  |  |  | Discovery set ^C^ | Replication set 1 ^C^ | Discovery set | Replication set 1 |
| *DCN* | 90104883 | rs10492230 | G/A | upstream | 0.18 | - | 0.15 | - |
|  | 90099338 | rs13312816^D^ | A/T | intron | 0.08 | 0.08 | 0.51 | 1.00 |
|  | 90098873 | rs741212 | A/G | intron | 0.13 | 0.13 | 0.37 | 0.89 |
|  | 90096865 | rs3138165^E^ | G/A | intron | 0.08 | 0.08 | 0.65 | 0.67 |
|  | 90081423 | rs516115 | A/G | intron | 0.29 | 0.30 | 0.23 | 0.17 |
| *LUM* | 90033280 | rs17018765^E^ | A/G | upstream | 0.08 | 0.08 | 0.36 | 0.67 |
|  | 90028976 | rs10859110 | G/A | intron | 0.26 | - | 0.67 | - |
|  | 90025329 | rs2268578 | G/A | intron | 0.14 | - | 0.01 | - |
|  | 90023166 | rs10745553 | C/G | intron | 0.16 | - | 0.71 | - |
|  | 90019294 | rs17714469 | G/A | downstream | 0.11 | - | 0.74 | - |
|  | 90016919 | rs1920790 | A/C | downstream | 0.14 | - | 0.79 | - |

^A^ Base pairs on chromosome 12 using genome build 36.2

^B^ MAF, Minor allele frequency; HWE, Hardy Weinberg Equilibrium

^C^ Discovery set: MAY and NCO; Replication set 1: AUS

^D^ Imputed in discovery set

^E^Imputed in replication set 1
