# Supplementary material for: Polymorphisms in Stromal Genes and Susceptibility to Serous Epithelial Ovarian Cancer: A Report from the Ovarian Cancer Association Consortium
Source: PLoS One. 2011 May 27;6(5):e19642. doi: 10.1371/journal.pone.0019642 (PMC3103497; doi:10.1371/journal.pone.0019642)
Supplement: Table S2 — Odds ratios (OR) and 95% confidence intervals (CI) for the association between genetic polymorphisms in DCN and LUM and serous epithelial ovarian cancer risk among 1,317 Caucasian subjects in the discovery set. (DOC) [file pone.0019642.s006.doc]

**Table S2. Odds ratios (OR) and 95% confidence intervals (CI) for the association between genetic polymorphisms in *DCN* and *LUM* and serous epithelial ovarian cancer risk among 1,317 Caucasian subjects in the discovery set^A^**

| Gene/SNP  rsID | Homozygous major allele (Referent) | | Heterozygous | | | Homozygous minor allele | | |  | Ordinal (Per minor allele) | |
| --- | --- | --- | --- | --- | --- | --- | --- | --- | --- | --- | --- |
|  | Cases | Controls | Cases | Controls | OR (95%CI)^B^ | Cases | Controls | OR (95%CI)^B^ | 2 df P-value | OR (95%CI)^B^ | P_trend_ |
| *DCN* |  |  |  |  |  |  |  |  |  |  |  |
| rs10492230 | 277 | 610 | 107 | 285 | 0.8 (0.6-1.1) | 10 | 10 | 1.0 (0.5-2.1) | 0.41 | 0.9 (0.7-1.1) | 0.27 |
| rs741212 | 313 | 699 | 81 | 210 | 0.9 (0.6-1.2) | 3 | 11 | 0.7 (0.2-2.4) | 0.53 | 0.9 (0.7-1.1) | 0.26 |
| rs3138165 | 353 | 778 | 43 | 138 | 0.7 (0.5-1.0) | 1 | 4 | 0.7 (0.1-6.2) | 0.17 | 0.7 (0.5-1.0) | 0.06 |
| rs516115 | 224 | 462 | 148 | 390 | 0.8 (0.6-1.0) | 23 | 67 | 0.7 (0.4-1.2) | 0.14 | 0.8 (0.7-1.0) | 0.06 |
| *LUM* |  |  |  |  |  |  |  |  |  |  |  |
| rs17018765 | 354 | 775 | 42 | 139 | 0.7 (0.5-1.0) | 1 | 3 | 0.9 (0.1-8.6) | 0.15 | 0.7 (0.5-1.0) | 0.06 |
| rs10859110 | 229 | 498 | 148 | 361 | 0.9 (0.7-1.2) | 18 | 60 | 0.7-0.4-1.2) | 0.33 | 0.9 (0.7-1.1) | 0.17 |
| rs2268578 | 303 | 671 | 88 | 239 | 0.8 (0.6-1.1) | 6 | 14 | 1.7 (0.6-4.9) | 0.20 | 0.9 (0.7-1.2) | 0.37 |
| rs10745553 | 278 | 649 | 109 | 245 | 1.0 (0.8-1.4) | 8 | 25 | 0.8 (0.3-1.7) | 0.77 | 1.0 (0.8-1.2) | 0.92 |
| rs17714469 | 321 | 720 | 71 | 188 | 0.9 (0.6-1.2) | 5 | 10 | 1.2 (0.4-3.5) | 0.60 | 0.9 (0.7-1.2) | 0.47 |
| rs1920790 | 307 | 677 | 87 | 226 | 0.9 (0.7-1.1) | 3 | 17 | 0.4 (0.1-1.4) | 0.24 | 0.8 (0.6-1.1) | 0.13 |

^A^ Discovery set: MAY and NCO

^B^ Adjusted for region of residence (Minnesota, Iowa, Wisconsin, Illinois, North Dakota, South Dakota and North Carolina)
