# Supplementary material for: Polymorphisms in Stromal Genes and Susceptibility to Serous Epithelial Ovarian Cancer: A Report from the Ovarian Cancer Association Consortium
Source: PLoS One. 2011 May 27;6(5):e19642. doi: 10.1371/journal.pone.0019642 (PMC3103497; doi:10.1371/journal.pone.0019642)
Supplement: Table S3 — Haplotype analysis of decorin and lumican genes and invasive serous epithelial ovarian cancer risk among 1,317 Caucasian subjects in the discovery set. (DOC) [file pone.0019642.s007.doc]

**Table S3. Haplotype analysis^A^ of decorin and lumican genes and invasive serous epithelial ovarian cancer risk among 1,317 Caucasian subjects in the discovery set^B^**

| Gene | Haplotype | Estimated haplotype frequency^C^ | Individual haplotype score^D^ | Individual haplotype test P-value |
| --- | --- | --- | --- | --- |
|  |  |  |  |  |
| *DCN*^E^ | AGGA | 7.21 | -2.03 | 0.04 |
|  | GGAG | 12.07 | -1.28 | 0.20 |
|  | GGAA | 5.31 | -0.34 | 0.73 |
|  | GGGA | 2.71 | 0.41 | 0.68 |
|  | GAGA | 72.65 | 2.16 | 0.03 |
| Global haplotype test P-value for decorin = 0.21 | | | | |
|  |  |  |  |  |
| *LUM*^F^ | GCAAGG | 7.07 | -1.91 | 0.06 |
|  | AAGACA | 10.52 | -0.86 | 0.39 |
|  | AAGAGA | 0.23 | -0.54 | 0.59 |
|  | GCGAGA | 0.54 | -0.48 | 0.63 |
|  | GCAAGA | 0.79 | -0.22 | 0.82 |
|  | GAGAGA | 0.27 | -0.20 | 0.84 |
|  | GCGGGA | 5.00 | 0.09 | 0.93 |
|  | GAAACA | 5.43 | 1.01 | 0.31 |
|  | GAGGGA | 69.83 | 1.33 | 0.18 |
| Global haplotype test P-value for lumican = 0.71 | | | | |

^A^ Adjusted for age (<40, 40-49, 50-59, 60-69 and 70+ yrs) and region of residence (Minnesota, Iowa, Wisconsin, Illinois, North Dakota, South Dakota and North Carolina)

^B^ Discovery set: MAY and NCO

^C^ Estimated using both cases and controls

^D^ Score statistic obtained by comparing haplotype of interest with all other haplotypes combined. A positive score indicates increased risk, while a negative score indicates decreased risk.

^E^ SNPs that formed haplotypes were rs3138165 (A/G), rs516115 (A/G), rs1049223 (G/A) and rs741212 (A/G)

^F^ SNPs that formed haplotypes were rs1771446 (G/A), rs1920790 (A/C), rs2268578 (G/A), rs10859110 (G/A), rs10745553 (G/C) and rs17018765 (A/G)
