# Supplementary material for: Polymorphisms in Stromal Genes and Susceptibility to Serous Epithelial Ovarian Cancer: A Report from the Ovarian Cancer Association Consortium
Source: PLoS One. 2011 May 27;6(5):e19642. doi: 10.1371/journal.pone.0019642 (PMC3103497; doi:10.1371/journal.pone.0019642)
Supplement: Table S4 — Genotype counts, MAF and HWE statistics and associations between genotypes and risk of ovarian carcinoma for DCN and LUM SNPs among Caucasian subjects in OCAC replication set 2. (DOC) [file pone.0019642.s008.doc]

**Table S4. Genotype counts, MAF and HWE statistics and associations between genotypes and risk of ovarian carcinoma for *DCN* and *LUM* SNPs among Caucasian subjects in OCAC replication set 2**

|  |  |  | AA^B^ | | Aa^B^ | | aa^B^ | | Ordinal | | |
| --- | --- | --- | --- | --- | --- | --- | --- | --- | --- | --- | --- |
| Site | MAF^A^ | HWE P-value^A^ | Ca | Co | Ca | Co | Ca | Co | OR | 95% CI | P trend |
| *DCN* rs13312816 | |  |  |  |  |  |  |  |  |  |  |
| BEL | 0.10 | 0.73 | 101 | 204 | 15 | 46 | 3 | 3 | 0.9 | 0.5-1.4 | 0.55 |
| GER | 0.06 | 0.61 | 79 | 230 | 20 | 33 | 1 | 0 | 1.9 | 1.1-3.4 | 0.03 |
| HAN-HJO | 0.06 | 0.91 | 52 | 377 | 9 | 46 | 1 | 2 | 1.5 | 0.8-3.0 | 0.21 |
| HAN-HMO | 0.03 | 0.51 | 65 | 137 | 9 | 8 | 0 | 1 | 1.8 | 0.7-4.3 | 0.22 |
| HOC | 0.05 | 1.00 | 126 | 390 | 8 | 42 | 0 | 1 | 0.6 | 0.3-1.2 | 0.16 |
| LAX | 0.08 | 1.00 | 125 | 125 | 30 | 23 | 4 | 1 | 1.5 | 0.9-2.4 | 0.16 |
| NTH | 0.07 | 0.05 | 84 | 285 | 13 | 37 | 1 | 4 | 1.1 | 0.6-2.0 | 0.73 |
| OVA | 0.07 | 0.24 | 220 | 399 | 31 | 60 | 1 | 0 | 1.0 | 0.6-1.6 | 0.99 |
| PVD | 0.07 | 0.61 | 128 | 187 | 25 | 28 | 1 | 0 | 1.4 | 0.8-2.5 | 0.24 |
| SOC | 0.05 | 1.00 | 134 | 350 | 30 | 39 | 0 | 1 | 1.9 | 1.1-3.1 | 0.02 |
| SRO | 0.06 | 0.78 | 203 | 820 | 27 | 110 | 0 | 4 | 0.9 | 0.6-1.4 | 0.72 |
| UCI | 0.08 | 0.05 | 91 | 211 | 26 | 31 | 2 | 4 | 1.6 | 1.0-2.6 | 0.06 |
| Pooled | 0.06 | 0.37 | 1408 | 3715 | 243 | 506 | 14 | 21 | 1.2 | 1.0-1.4 | 0.02 |
| *DCN* rs3138165 | |  |  |  |  |  |  |  |  |  |  |
| BEL | 0.10 | 0.73 | 101 | 204 | 15 | 45 | 3 | 3 | 0.9 | 0.5-1.5 | 0.59 |
| GER | 0.07 | 0.61 | 78 | 230 | 20 | 35 | 1 | 0 | 1.8 | 1.0-3.3 | 0.04 |
| HAN-HJO | 0.06 | 0.91 | 52 | 378 | 9 | 46 | 1 | 2 | 1.5 | 0.8-3.0 | 0.21 |
| HAN-HMO | 0.03 | 0.49 | 65 | 137 | 9 | 8 | 0 | 1 | 1.8 | 0.7-4.3 | 0.22 |
| HOC | 0.05 | 0.61 | 126 | 390 | 9 | 43 | 0 | 0 | 0.6 | 0.3-1.4 | 0.26 |
| LAX | 0.09 | 1.00 | 125 | 126 | 32 | 24 | 3 | 1 | 1.4 | 0.8-2.4 | 0.19 |
| NTH | 0.07 | 0.19 | 84 | 285 | 14 | 39 | 1 | 3 | 1.2 | 0.7-2.1 | 0.58 |
| OVA | 0.07 | 0.24 | 219 | 400 | 32 | 60 | 1 | 0 | 1.0 | 0.7-1.6 | 0.87 |
| PVD | 0.07 | 0.61 | 127 | 187 | 25 | 28 | 2 | 0 | 1.5 | 0.9-2.6 | 0.15 |
| SOC | 0.05 | 0.61 | 134 | 352 | 31 | 40 | 0 | 0 | 2.0 | 1.2-3.4 | 0.01 |
| SRO | 0.06 | 0.79 | 203 | 819 | 27 | 112 | 0 | 4 | 0.9 | 0.6-1.4 | 0.66 |
| UCI | 0.08 | 0.06 | 92 | 211 | 25 | 32 | 2 | 4 | 1.5 | 0.9-2.5 | 0.09 |
| Pooled | 0.06 | 0.90 | 1406 | 3719 | 248 | 512 | 14 | 18 | 1.2 | 1.1-1.4 | 0.01 |
| *DCN* rs516115 | |  |  |  |  |  |  |  |  |  |  |
| BEL | 0.33 | 0.78 | 67 | 113 | 41 | 114 | 11 | 26 | 0.7 | 0.5-1.0 | 0.09 |
| GER | 0.27 | 0.88 | 51 | 140 | 33 | 103 | 14 | 20 | 1.2 | 0.8-1.7 | 0.31 |
| HAN-HJO | 0.26 | 0.89 | 34 | 230 | 23 | 164 | 5 | 30 | 1.0 | 0.7-1.5 | 0.96 |
| HAN-HMO | 0.24 | 0.51 | 41 | 79 | 27 | 55 | 6 | 7 | 1.1 | 0.7-1.7 | 0.67 |
| HOC | 0.21 | 1.00 | 88 | 268 | 40 | 146 | 6 | 19 | 0.9 | 0.6-1.3 | 0.52 |
| LAX | 0.29 | 1.00 | 77 | 75 | 68 | 62 | 14 | 13 | 1.0 | 0.7-1.5 | 0.82 |
| NTH | 0.26 | 0.19 | 43 | 185 | 45 | 115 | 11 | 26 | 1.5 | 1.0-2.0 | 0.03 |
| OVA | 0.28 | 0.82 | 141 | 239 | 87 | 184 | 24 | 37 | 0.9 | 0.7-1.2 | 0.62 |
| PVD | 0.23 | 0.35 | 79 | 128 | 63 | 72 | 12 | 14 | 1.3 | 0.9-1.8 | 0.14 |
| SOC | 0.27 | 0.37 | 79 | 206 | 71 | 162 | 15 | 24 | 1.2 | 0.9-1.6 | 0.19 |
| SRO | 0.27 | 0.12 | 122 | 481 | 96 | 391 | 12 | 60 | 0.9 | 0.7-1.2 | 0.56 |
| UCI | 0.27 | 0.87 | 56 | 129 | 49 | 96 | 14 | 19 | 1.3 | 0.9-1.8 | 0.18 |
| Pooled | 0.27 | 0.67 | 878 | 2273 | 643 | 1667 | 144 | 295 | 1.1 | 1.0-1.2 | 0.29 |
| *LUM* rs17018765 | |  |  |  |  |  |  |  |  |  |  |
| BEL | 0.10 | 0.28 | 100 | 207 | 16 | 42 | 3 | 4 | 0.9 | 0.6-1.5 | 0.79 |
| GER | 0.07 | 0.61 | 77 | 230 | 21 | 35 | 1 | 0 | 1.9 | 1.1-3.4 | 0.03 |
| HAN-HJO | 0.06 | 0.68 | 52 | 380 | 9 | 44 | 1 | 2 | 1.6 | 0.8-3.1 | 0.17 |
| HAN-HMO | 0.04 | 0.61 | 63 | 135 | 11 | 10 | 0 | 1 | 1.8 | 0.8-4.2 | 0.15 |
| HOC | 0.05 | 0.36 | 125 | 388 | 10 | 43 | 0 | 2 | 0.7 | 0.3-1.3 | 0.27 |
| LAX | 0.08 | 1.00 | 131 | 128 | 26 | 23 | 3 | 0 | 1.3 | 0.8-2.3 | 0.30 |
| NTH | 0.06 | 0.14 | 85 | 286 | 13 | 36 | 1 | 3 | 1.2 | 0.7-2.1 | 0.60 |
| OVA | 0.06 | 0.24 | 222 | 400 | 30 | 59 | 0 | 0 | 0.9 | 0.6-1.5 | 0.72 |
| PVD | 0.07 | 0.61 | 126 | 187 | 27 | 28 | 1 | 0 | 1.5 | 0.9-2.7 | 0.14 |
| SOC | 0.05 | 1.00 | 134 | 352 | 31 | 40 | 0 | 1 | 1.9 | 1.2-3.1 | 0.01 |
| SRO | 0.06 | 1.00 | 201 | 823 | 27 | 108 | 0 | 3 | 1.0 | 0.6-1.5 | 0.88 |
| UCI | 0.07 | 0.02 | 96 | 213 | 21 | 27 | 2 | 4 | 1.4 | 0.9-2.4 | 0.15 |
| Pooled | 0.06 | 0.43 | 1412 | 3729 | 242 | 496 | 12 | 20 | 1.2 | 1.1-1.4 | 0.01 |

^A^ MAF, minor allele frequency among control subjects; HWE P, P value testing Hardy-Weinberg equilibrium among control subjects; Ca, cases; Co, controls

^B^ Referent is no copies of the minor allele (AA); Aa, one copy of the minor allele; aa, two copies of the minor allele
