# Supplementary material for: Polymorphisms in Stromal Genes and Susceptibility to Serous Epithelial Ovarian Cancer: A Report from the Ovarian Cancer Association Consortium
Source: PLoS One. 2011 May 27;6(5):e19642. doi: 10.1371/journal.pone.0019642 (PMC3103497; doi:10.1371/journal.pone.0019642)
Supplement: Table S5 — Overview of 18 OCAC studies with serous epithelial ovarian cancer cases and controls. (DOC) [file pone.0019642.s009.doc]

**Table S5. Overview of 18 OCAC studies with serous epithelial ovarian cancer cases and controls**

|  |  | **Cases** | | | **Controls** | | |
| --- | --- | --- | --- | --- | --- | --- | --- |
| **Study** | **Design** | **N** | **Age** | **Ascertainment** | **N** | **Age** | **Ascertainment** |
| **Discovery set** | | | | | | | |
| MAY (USA) | Clinic-based | 191 | >20 | Cases attending Mayo Clinic diagnosed from 2000 onwards identified in a six state surrounding region (Minnesota, Iowa, Wisconsin, Illinois, North Dakota and South Dakota). Response was 83%. | 439 | >20 | Identified through Mayo Clinic. Women seeking general medical examination. Frequency matched to cases on age and region of residence. Response was 74%. |
| NCO (USA) | Population-based | 206 | 20-74 | Cases from 1999 onwards identified from 48 counties within North Carolina by rapid-case ascertainment. Response was 75% among eligible cases. | 481 | 22-75 | Controls selected using random digit dialing and matched by age (5-year categories) and race (black vs. non-black). Response was 64% among eligible controls (54% among controls who could be contacted). |
| **Replication set 1** | | | | | | | |
| AUS (Australia) | Population-based | 436 | 19-79 | Diagnosed from 2002 onwards; recruited through surgical treatment centers throughout Australia & cancer registries of Queensland S. Australia & W. Australia (AOCS) & cancer registries of New South Wales & Victoria (ACS). Response was 84%. | 1,098 | 19-79 | Randomly selected from Commonwealth electoral roll. Frequency matched for age and geographical region. Response was 47%. |
| **Replication set 2** | | | | | | | |
| BEL (Belgium) | Hospital-based | 119 | 14-85 | Cases attending the Gynecologic Oncology Unit at the Leuven University Hospital diagnosed with incident ovarian cancer from 2009 onwards. Response was 95%. | 252 | 19-66 | Randomly selected healthy blood donors frequenting the Blood Transfusion Center in the University Hospital of Leuven with matched Flemish/Belgian ethnicity to the ovarian cancer cases. Response was 95%. |
| GER (Germany) | Population-based | 99 | 20-75 | Incident cases diagnosed 1993 -1996 from two study areas in southern Germany and identified through frequent monitoring of hospitals serving the study areas. Response was 58%. | 265 | 23-75 | Two controls per case matched by age and study area were selected from a random sample of the general female population in study area selected using population registries. Response was 51%. |
| HAN-HJO (Germany) | Hospital-based | 62 | 18-88 | Hospital based cases from the Gynecological Departments at the Friedrich-Schiller University of Jena, recruited between 1999-2009, and at Hannover Medical School, recruited from 2007-2009. Not actively selected for family history, early onset, or histology. Response was 75%. | 426 | 18-68 | Female controls recruited from randomly selected healthy German blood bank donors at Hannover Medical School with matched ethnicity to the ovarian cancer cases. Response was 70%. |
| HAN-HMO (Germany) | Hospital-based | 74 | 24-76 | Hospital based cases from the N.N Aleksandrov University of Minsk, Belarus, recruited between 2006-2009. Not actively selected for family history, early onset, histology or other specific criteria. Response was 60%. | 146 | 22-71 | Female controls recruited from healthy Byelorussian volunteers at the N.N Aleksandrov University of Minsk, Belarus. Selected for absence of personal cancer history and no family history of breast or ovarian cancer. Response n/a. |
| HOC (Finland) | Population-based | 135 | n/a | Cases treated between 1989-2003 at the Department of Obstetrics and Gynecology of the Helsinki University Central Hospital, where treatment of ovarian cancer patients from Southern Finland is centralized. Response n/a. | 433 | 18-65 | Healthy women from the same geographical region in Southern Finland. Response n/a. |
| LAX (USA) | Hospital-based | 160 | ≥18 | Cases are identified through the Women's Cancer Research Institute biorepository from 1989 onwards. Patients presenting to the gynecologic cancer service with epithelial ovarian cancer are identified in IRB 901 (Tissue Bank) and IRBs 1080 and 4049 (Gilda Radner Hereditary Cancer ). Response n/a. | 151 | 18-85 | UCI excess controls – see UCI study |
| MAL (Denmark) | Population-based | -- | -- | -- | 215 | 35-79 | Random selection of females from the computerized Central Population Register. Response was 67%. |
| NTH (Netherlands) | Population-based | 99 | 11-83 | Prevalent ovarian cancers diagnosed between 1989 and 2006 and still alive in 2008, identified from the population-based cancer registry of the Comprehensive Cancer Centre East in the Mid-Eastern part of the Netherlands. Response was 68%. | 327 | 30-75 | An age-stratified random sample of the general population of the municipality of Nijmegen in 2002, frequency age-matched to the cases. Response was 43%. |
| OVA (Canada) | Population-based | 252 | 20-79 | Cases diagnosed 2002-2007 identified through the population-based British Columbia Cancer Registry. Response was 64%. | 460 | 20-79 | Controls identified through the British Columbia population-based provincial health roster and frequency matched to cases on age (5-yr). Response was 49%. |
| PVD (Denmark) | Hospital-based | 154 | 17-89 | All patients admitted with a pelvic mass at Rigshospitalet, University of Copenhagen, between 2004 and 2009 are included in the study with a blood sample less than 14 days before surgery/diagnosis of ovarian cancer and with FFPE and fresh frozen tissues if possible. Response was 90%. | 215 | 35-79 | MAL controls – see MAL study |
| SEA (UK) | Population-based | -- | -- | -- | 670 | 39-77 | Selected from the EPIC-Norfolk cohort of 25000 apparently healthy individuals recruited between 1993 and 1997 from one of 121 General Practitioners based in the same geographical regions as the cases. Response was 84%. |
| SOC (UK) | Hospital-based | 165 | 23-92 | SOC cases represent a sequential series of patients presumed to be Caucasian undergoing primary surgery or epithelial ovarian cancer at hospitals in the Wessex region of Southern England between 1993 and 1998. Response n/a. | 392 | 50-76 | 392 UKO controls –see UKO study |

| SRO (UK) | Hospital-based/Clinical trial | 230 | 18-84 | Mostly Caucasian patients randomized into a prospective phase III comparison of paclitaxel-carboplatin versus docetaxel-carboplatin as first line chemotherapy in stage Ic-IV epithelial ovarian cancer (SCOTROC 1) which recruited from 1998 to 2000. Response was 81%. | 935 | -- | 265 UKO and 670 SEA controls – see UKO and SEA studies |
| --- | --- | --- | --- | --- | --- | --- | --- |

| UKO (UK) | Population-based | -- | -- | -- | 657 | 50-76 | Apparently healthy postmenopausal women from the general population participating in the United Kingdom Collaborative Trial of Ovarian Cancer Screening (UKCTOCS). All women followed up for cancers through the NHS Information Centre for Health and Social Care (formerly the Office of National Statistics. Response was 97%. |
| --- | --- | --- | --- | --- | --- | --- | --- |
| UCI (USA) | Population-based | 119 | 18-85 | Rapid cases ascertainment through Orange and San Diego Cancer Surveillance program from 1993-2003. Response was 80%. | 247 | 18-85 | Random-digit dial identification from study area. Frequency matched to cases for race/ethnicity & 5 year age group. Response was 70%. |
