# Supplementary material for: Polymorphisms in Stromal Genes and Susceptibility to Serous Epithelial Ovarian Cancer: A Report from the Ovarian Cancer Association Consortium
Source: PLoS One. 2011 May 27;6(5):e19642. doi: 10.1371/journal.pone.0019642 (PMC3103497; doi:10.1371/journal.pone.0019642)
Supplement: Text S1 — Ethics statement. (DOC) [file pone.0019642.s010.doc]

**Text S1**

**Ethics statement.** Participants in all the studies provided written informed consent and each site’s institutional review board approved the study protocol, including Ethics Committees of the Queensland Institute of Medical Research and Peter MacCallum Cancer Centre (AUS); the local Ethical Committee (Commissie Medische Ethiek UZ Leuven, Belgium) (BEL); Ethics Committee of the University of Heidelberg (GER); the National Supervisory Authority of Welfare and Health (HOC); Ethical Review Board at Hannover Medical School (HJO, HMO); Ethics Commission of the State Organization ‘Institute for Hereditary Diseases', Ministry of Health, Republic of Belarus (HMO); Institutional Review Board of Cedars-Sinai Medical Center (LAX); Danish Central Scientific Ethical Committee (MAL); Institutional Review Board of Mayo Clinic (MAY); Institutional Review Board of Duke University Medical Center (NCO); Institutional Review Board of the University Medical Centre Nijmegen (NTH); University of British Columbia-British Columbia Cancer Agency Research Ethics Board (OVA); Scientific Ethical Committee for the Capital Region (PVD); Cambridgeshire 4 Research Ethics Committee (SEA); Southampton and South West Hampshire Research Ethics Committee (SOC); Multi-Centre Research Ethics Committee for Scotland (SRO); University of California, Irvine Institutional Review Board (UCI); and the National Health Service Central Office for Research Ethics Committees (COREC) and The Joint University College London/University College London Hospital Committee on the Ethics of Human Research (Committee A) (UKO).
